# Investigation of the impact of bromodomain inhibition on cytoskeleton stability and contraction

**DOI:** 10.1186/s12964-024-01553-6

**Published:** 2024-03-16

**Authors:** Alexander Bigger-Allen, Ali Hashemi Gheinani, Rosalyn M. Adam

**Affiliations:** 1 Urological Diseases Research Center, Boston Children’s Hospital, Enders Bldg 1061.4, 300 Longwood Avenue, Boston, MA 02115 USA; 2 Biological & Biomedical Sciences Program, Division of Medical Sciences, Harvard Medical School, Boston, MA USA; 3 Department of Surgery, Harvard Medical School, Boston, MA USA; 4 Functional Urology Research Group, Department for BioMedical Research DBMR, University of Bern, Bern, Switzerland; 5 Department of Urology, Inselspital University Hospital, 3010 Bern, Switzerland; 6 Broad Institute of MIT and Harvard, Cambridge, MA USA

**Keywords:** PDGF, TGFβ, Myc, JQ1, Smooth muscle cells, Fibroblasts

## Abstract

**Background:**

Injury to contractile organs such as the heart, vasculature, urinary bladder and gut can stimulate a pathological response that results in loss of normal contractility. PDGF and TGFβ are among the most well studied initiators of the injury response and have been shown to induce aberrant contraction in mechanically active cells of hollow organs including smooth muscle cells (SMC) and fibroblasts. However, the mechanisms driving contractile alterations downstream of PDGF and TGFβ in SMC and fibroblasts are incompletely understood, limiting therapeutic interventions.

**Methods:**

To identify potential molecular targets, we have leveraged the analysis of publicly available data, comparing transcriptomic changes in mechanically active cells stimulated with PDGF and TGFβ. Additional Analysis of publicly available data sets were performed on SMC and fibroblasts treated in the presence or absence of the MYC inhibitor JQ1. Validation of in silico findings were performed with qPCR, immunoblots, and collagen gel contraction assays measure the effect of JQ1 on cytoskeleton associated genes, proteins and contractility in mechanically active cells. Likelihood ratio test and FDR adjusted *p*-values were used to determine significant differentially expressed genes. Student ttest were used to calculate statistical significance of qPCR and contractility analyses.

**Results:**

Comparing PDGF and TGFβ stimulated SMC and fibroblasts identified a shared molecular profile regulated by MYC and members of the AP-1 transcription factor complex. Additional in silico analysis revealed a unique set of cytoskeleton-associated genes that were sensitive to MYC inhibition with JQ1. In vitro validation demonstrated JQ1 was also able to attenuate TGFβ and PDGF induced changes to the cytoskeleton and contraction of smooth muscle cells and fibroblasts in vitro.

**Conclusions:**

These findings identify MYC as a key driver of aberrant cytoskeletal and contractile changes in fibroblasts and SMC, and suggest that JQ1 could be used to restore normal contractile function in hollow organs.

**Supplementary Information:**

The online version contains supplementary material available at 10.1186/s12964-024-01553-6.

## Introduction

The function of hollow organs such as the heart, gut, vasculature and urinary bladder rely on coordinated cycles of contraction and relaxation. In response to injury, the normal contractile activity of mechanically active cells such as smooth muscle cells, fibroblasts and myofibroblasts, is perturbed leading to aberrant contraction and functional decline [1]. In addition to actomyosin cross-bridge cycling, tension generation in cells involves contributions from the actin cytoskeleton, as evidenced by the reduction in smooth muscle contraction following pharmacological inhibition of actin polymerization (reviewed in [2]). In spite of extensive study of mechanisms that regulate the actin cytoskeleton, exploiting this knowledge to reverse aberrant contraction remains challenging.

Previous studies from our group identified the AP-1 transcriptional complex as a key regulator of smooth muscle cell behavior in response to discrete stimuli such as cyclic stretch-relaxation and the physiologically relevant growth factors Transforming Growth Factor beta (TGFβ) and Platelet Derived Growth Factor (PDGF) [3–8]. TGFβ, a known inducer of smooth muscle differentiation and activator of fibroblast to myofibroblast transdifferentiation [9, 10] was found to induce the formation of filamentous actin in smooth muscle cells [7]. In that study, knockdown of the AP-1 monomer JUNB decreased both basal and TGFβ-stimulated cell contraction and cytoskeletal tension, in parallel with reduction in phosphorylation of both Myosin Light Chain 20 (MLC20) and cofilin (CFL). Of note, these changes occurred independently of alterations in the SM markers; alpha Smooth Muscle Actin (αSMA), Smooth Muscle protein 22 (SM22α) and Calponin 1 (CNN1). We also implicated Activator Protein 1 (AP-1) in regulation of gene expression and Smooth muscle cell (SMC) phenotype downstream of Platelet Derived Growth Factor Receptor (PDGFR) -mediated signaling [5, 8]. In that study an unbiased assessment of PDGF-induced transcriptomic changes in SMC identified AP-1 and MYC proto-oncogene (MYC) as the transcriptional regulators most highly linked to up-regulated genes [8]. In addition, integration of gene expression data with proteomics led to the identification of a novel MYC-centric network in SMC. Moreover, manipulation of the MYC target and RHOA effector DIAPH3, by siRNA or with a MYC inhibitor, led to marked cytoskeletal changes in SMC consistent with prior reports linking DIAPH3/mDia2 to RHOA-dependent regulation of SMC phenotype [11, 12].

Previous studies have explored the functional relationship between MYC and actin cytoskeleton regulation in non-muscle cells [13–18]. Whereas some reports have identified MYC as an inhibitor of actin filament polymerization [15], others have demonstrated enhanced formation of F-actin stress fibers [18]. However, since these analyses were conducted under conditions where MYC was overexpressed, the significance of the functional relationship between MYC and cytoskeletal regulation in non-transformed cells with endogenous MYC expression was unclear. To address this gap in knowledge, a number of groups have explored the impact of pharmacological inhibition of endogenous MYC. Among the inhibitors assessed, JQ1 has been shown to attenuate MYC expression indirectly by preventing the bromo and extra-terminal (BET) family of proteins from binding to acetylated histones [19]. Although studied primarily in the context of MYC overexpression in cancer, JQ1 and other BET protein inhibitors have also been employed in non-cancer settings including fibrosis and cardiac diseases. In these studies JQ1 treatment attenuated epithelial-to-mesenchymal transition, migration and activation of myofibroblasts consistent with an impact on the cytoskeleton [20]. However, the extent to which inhibiting MYC may be beneficial in settings of aberrant contraction remains unclear. In this study, we have identified MYC as a central mediator of pathophysiologically relevant stimuli in smooth muscle cells and fibroblasts. Additionally, we have explored the impact of MYC inhibition on the regulation of the actin cytoskeleton and contraction in these mechanically active cells.

## Results

### In silico analysis reveals common transcriptional regulators in mechanically active cells

**Fig. 1:**
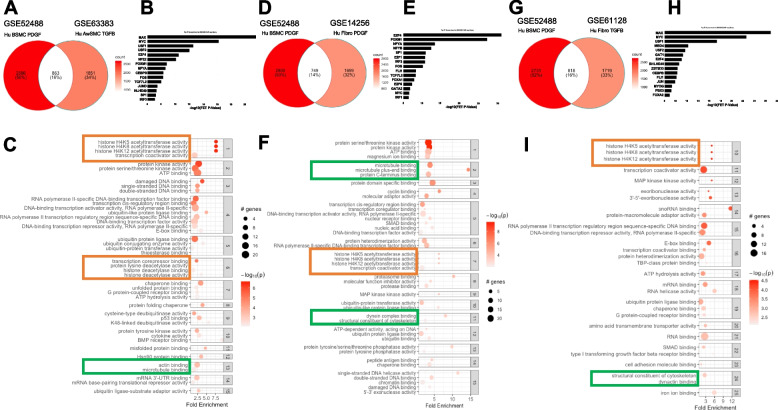
Comparative analysis of growth factor stimulated mechanically active cells identifies common TF regulators and changes in chromatin and the cytoskeleton. **A**, **D**, **G** Venn diagrams comparing our PDGF stimulated pHBSMC DEGs to the DEGs generated from reanalyzed publically available data of smooth muscle cells or fibroblasts stimulated with PDGF or TGFB. **B**, **E**, **H** Transcription factor master regulator analysis using CHEA3 and ENCODE database of all the shared DEGs from A, D, and G identified MYC, MAX and members of the AP-1 transcription factor complex as the most highly networked regulators based on -log Fisher's exact test (FET) *p*-value. **C**, **F**, **I** Shared DEGs from A, D, and G were subjected to enrichment analysis using GO terms for molecular function. Terms were then clustered to group molecular functions with overlapping DEGs. Highlighted in orange, are molecular functions related to chromatin remodeling. Highlighted in green are molecular functions related to cytoskeleton regulation

Prior findings from our groups implicated PDGF and TGFβ as drivers of key pathways in SMC relevant to pathological tissue remodeling [5–8, 20, 21]. MYC and AP-1 emerged as functionally relevant mediators, although the extent to which they were regulated in common by PDGF and TGFβ remains unknown. To investigate effectors shared between PDGF- or TGFβ- treated SMC and fibroblasts, we re-analyzed gene expression data generated previously by us [8], and compared the results with re-analyzed publicly available data of SMC and fibroblasts stimulated with PDGF and TGFβ. We identified 8 microarray datasets, including our own, in which mechanically active cells were stimulated for similar, short durations with either cytokine, i.e. not exceeding 24 h (Supplemental Table 1). Only four of the eight datasets showed separation of the control and experimental group replicates by PCA plot and were utilized for differential expression analysis (Supplementary Fig. 1A-D). Differential gene expression using linear models for microarray analysis identified > 2000 differentially expressed genes in the publicly available datasets (Supplemental Fig. 2A, B, C). Comparative analyses identified nearly 800 DEGs in common between the datasets (Fig. 1A, D, and G). To better understand the regulatory network evoked by PDGF and TGFβ treatment, we used the ChEA3 transcription factor (TF) enrichment analysis tool [22] to identify TFs that could explain the gene expression profiles. The list of shared DEGs from each comparison was submitted to the ChEA3 online tool and compared against the ENCODE library as the reference for TF-target gene interactions. This database was generated from experimentally determined TF-gene interactions using ChIP-seq. The enriched TFs were rank ordered based on Fisher’s exact test (FET) and the top 10 were plotted based on the FET values (Fig. 1B, E, and H). This analysis identified MYC and the MYC-associated factor X (MAX) among the most enriched TFs, regulating the three gene sets of roughly 800 genes from each comparison. A second TF enrichment analysis was performed using the DAVID based interaction enrichment tool which accesses the UCSC Transfac Binding Site database (TFBS). Rank-ordering of TFs based on percentage of genes regulated from each shared DEG list identified MYCMAX (Supplemental Fig. 2D). Further, additional highly-enriched TFs from both analyses included members of the AP-1 complex such as FOS, FOSB, JUN and/or JUND, and AP-1 itself.

**Fig. 2:**
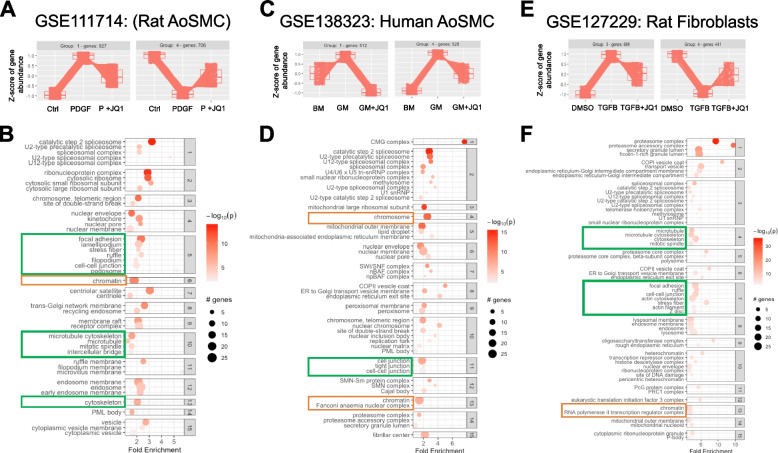
Pattern and enrichment analyses support JQ1 attenuation of genes associated with cytoskeletal changes. Four publicly available datasets in which mechanically active cells were stimulated with a physiologically relevant pro-fibrotic condition in the absence or presence of JQ1 were identified and reanalyzed. Differential gene expression analysis was performed using DESeq2 after generating count matrices from the fastq files using Biogrids software tools including: SRAtoolkit, STAR, Subread. **A**, **C**, **E** Pattern analysis of DEGs from each of the four datasets identified 2 groups of genes that were perturbed by PDGF (GSE11714), Growth medium (GSE138323), or TGFB (GSE127229) and attenuated by JQ1. **B**, **D**, **F** JQ1 sensitive genes were subjected to GO cellular compartment terms and clustered based on common genes between terms. Highlighted in yellow are terms related to chromatin remodeling. Highlighted in green are terms related to cytoskeleton regulation

To understand what processes the genes from each of the three shared DEG sets are involved in, we utilized two approaches for enrichment analysis. The first approach was a standard gene set enrichment analysis for each of the shared DEG lists using gene ontology (GO) terms for molecular function (MF), biological process (BP) and cellular compartment (CC) which were rank-ordered by the ratio of genes represented (Supplemental Figs. 3A, 4A, 5A). Due to the small number of DEGS in each list (~ 800), statistically significant enrichment of terms was limited. The GSEA curves for 3 to 4 representative enriched terms from each type of GO analysis were plotted (Supplemental Figs. 3, 4 and 5). To overcome the limitations of the standard GSEA approach, we utilized active subnetwork searches against the BIOGRID protein–protein interaction network database and subsequent enrichment analysis using the pathfindR package. Gene ontology (GO) terms for molecular function (MF) (Fig. 1C, F and I), cellular compartment (CC) (Supplementary Fig. 6A, B, and C), and biological process (BP, data not shown) were used to perform the enrichment analyses. Following the active subnetwork search and GO term enrichment, the terms were clustered using hierarchical clustering to group similar terms. In the first comparison, our previously generated data were compared to SMC stimulated with TGFβ. The top terms enriched downstream of shared DEGs from PDGF- and TGFβ-treated SMC include histone acetyltransferase and deacetylase activity in clusters 1 and 6 (7.66-fold enrichment, *p*-value = 9.29e-05 and 1.33–3.51-fold enrichment, *p*-value = 0.005 – 1.27e-05 respectively) (Fig. 1C). Similarly, enrichment analysis of shared DEGs between our dataset and GSE14256, in which fibroblasts were stimulated with PDGF, identified histone acetyltransferase activity in cluster 7 (6.26-fold enrichment, *p*-value = 4.31e-05) (Fig. 1F). Further, histone acetyltransferase activity was also identified in cluster 10 when comparing our data with GSE61128 in which fibroblasts are stimulated with TGFβ (5.02-fold enrichment, *p*-value = 0.0063) (Fig. 1I). The enrichment of histone acetyltransferase activity in all comparisons supports a similar perturbation of chromatin-related genes by both PDGF and TGFβ in SMC and fibroblasts.

**Fig. 3:**
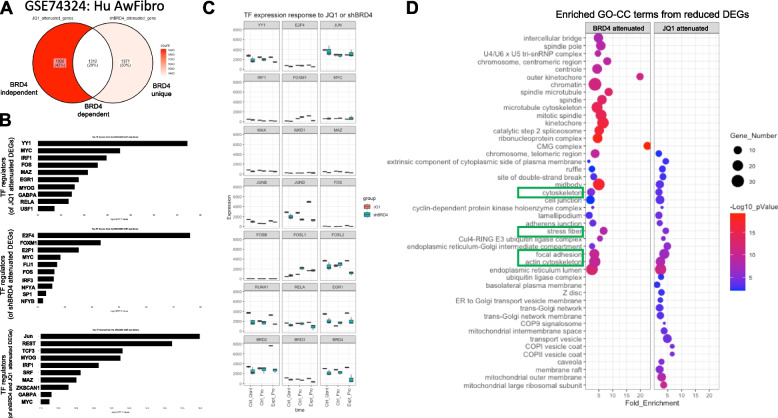
JQ1 and BRD4 knock-down converge on Myc and AP1 to attenuate cytoskeleton related genes. An additional dataset comprising IMR90, Human airway fibroblasts, under conditions of quiescence or growth and stimulated with DMSO, JQ1, control shRNA, or shBRD4. Pattern analysis was performed to identify JQ1 sensitive and BRD4 sensitive DEGs defined as those that were perturbed by growth conditions and attenuated to near baseline, quiescent levels with JQ1 or shBRD4. **A** A Venn diagram illustrating the extent of shared DEGs that were sensitive to both JQ1 and shBRD4. **B** Genes from each group in the Venn diagram; attenuated only by JQ1, attenuated only by shBRD4, and those attenuated by both were subjected to TF enrichment analysis with ChEA3 using the ENCODE, ChIP-seq database of TF-gene interactions was used to identify TFs implicated in regulated each group of genes. TFs were rank-ordered by the –log10 of the Fisher’s Exact Test (FET) value. **C** Normalized counts of relevant TFs implicated in the TF enrichment analysis, TFs implicated in previously published findings as well as bromodomains were plotted. Each plot contains the average expression of DMSO-stimulated quiescent cells (Ctrl_Qsnt), DMSO or shRNA control stimulated proliferative cells (Ctrl_Pro), and JQ1 or shBRD4 stimulated proliferative cells (Expt_Pro) in pink and blue respectively. JQ1 attenuated DEGs and shBRD4 attenuated DEGs were separated between up regulated and down regulated genes (based on their expression in JQ1 and shBRD4 proliferative cells compared to the proliferative control cells). The down regulated, JQ1 and shBRD4 genes were subjected to active subnetwork searches to BIOGRID protein–protein interaction network and subsequent enrichment analysis using GO terms for cellular compartment with the pathfindR package. **D** The shared and uniquely enriched terms between the shBRD4 (BRD4) attenuated DEGs and the JQ1 attenuated DEGs were plotted in a dotplot. Each dot reflects an enriched term, the x-axis reflects the fold enrichment of each term, the color of each dot corresponds to the –log10 of the adjusted *p*-value which is adjusted based on Bonferroni method and the size of each dot corresponds to the number of genes from the DEG lists associated with each term. Cytoskeleton associated terms sensitive to both JQ1 and BRD4 knock-down are highlighted with green rectangles

**Fig. 4:**
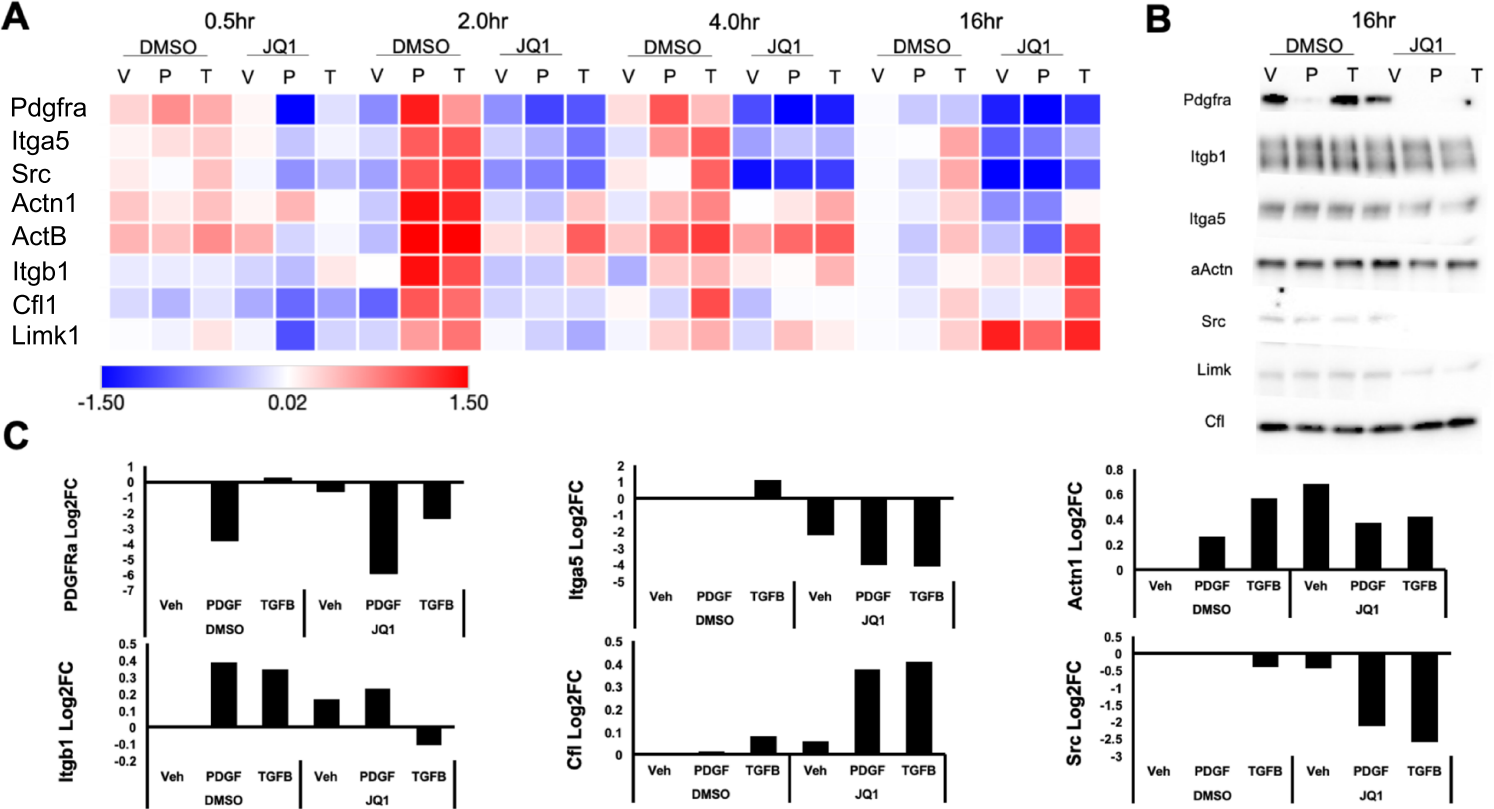
Validation of JQ1 sensitive cytoskeletal effectors via qPCR and immunoblots. A subset of cytoskeleton associated targets predicted to be sensitive to JQ1 were validated using qPCR and immunoblotting techniques. Primers for JQ1 sensitive cytoskeleton associated targets were generated using NCBI primer design tool. A time course of vehicle (V), PDGF (P), or TGFB (T) stimulated RBMC with concurrent stimulation of DMSO or JQ1 was performed. **A** The average Log2FC of three replicates was plotted in a heat map. **B** The same targets were assessed by immunoblot at 16 h and **C** quantified. Data are representative of 3 independent trials

**Fig. 5:**
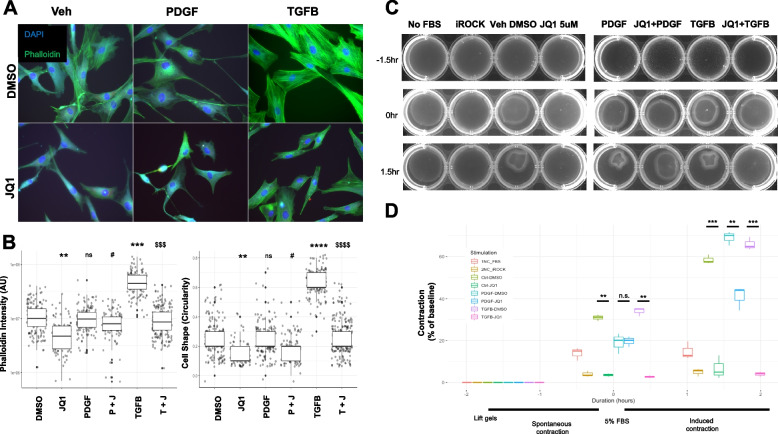
In vitro functional validation of JQ1 attenuation of PDGF and TGFB stimulated contraction in RBMC. **A** Representative images of RBMC stimulated with PBS (Veh), PDGF, TGFB with DMSO or JQ1 for 16 h and stained with Vimentin, Phalloidin and DAPI. **B** Phalloidin intensity and cell shape was quantified in ImageJ. **C** RBMC were plated on 1.2 mg/mL collagen gels in a 12 well plate in 1 mL of serum free media and incubated for 16 h with PBS (Veh), PDGF, TGFB with DMSO or JQ1. Select wells were stimulated for 30 min with a ROCK inhibitor as a negative control for contraction. Collagen gels were separated from the walls of the wells and Imaged after 1.5 h to capture spontaneous (unstimulated contraction, followed by an additional 1.5 h in 5% FBS to facilitate contraction. **D** Contraction was quantified using ImageJ and measured as a percent of the changed area of the gel from the baseline. Data are representative of 3 independent trials. 1NC_FBS refers to negative control for FBS in which cells never receive FBS from platting to harvesting. 2NC_iROCK refers to the negative control for contraction in which cells are stimulated with a ROCK inhibitor for 30 min prior to observing contraction. *, *p* < 0.05, ** *p* < 0.01, ***, *p* < 0.001, ****, *p* < 0.0001 compared to control. #, *p* < 0.05, ## *p* < 0.01, ###, *p* < 0.001, ###, *p* < 0.0001 compared to PDGF. $, *p* < 0.05, $$ *p* < 0.01, $$$, *p* < 0.001, $$$$, *p* < 0.0001 compared to TGFB. Statistical significance was calculated with student t-test

In all three comparisons of our data with fibroblasts and SMC stimulated with TGFβ or PDGF, the perturbation of genes associated with the cytoskeleton was also notable. To understand the extent to which all four datasets shared DEGs, we compared the DEGs across datasets and identified 89 shared genes (Supplementary Fig. 7A). Using the ChEA3 TF enrichment tool, MYC and MAX were predicted as the top most networked TF regulators (Supplementary Fig. 7B). Finally, gene set enrichment analysis using active subnetworks was performed using terms for MF, CC and BP terms (Supplementary Fig. 7C, D, E). In the CC enrichment analysis, positive regulation of cytoskeleton organization was one of the most enriched terms, comprising 5 genes (LIMK1, BIN1, CDKN1B, BCAS3, and WASL, gene list not shown) shared between all 4 datasets. Taken together these analyses implicate MYC and MAX as mediators of PDGF- and TGFβ-induced transcriptional changes to the cytoskeleton.

### Transcriptomic data from JQ1 treated mesenchymal cells support JQ1 attenuation of cytokine induced changes

Given the identification of MYC and MAX as highly enriched TFs downstream of both PDGF and TGFβ treatment, we next explored the impact of JQ1 treatment on gene expression profiles from mechanically active cells exposed to these same proliferative or pro-contractile stimuli. JQ1 is a bromodomain inhibitor that has been shown previously to inhibit MYC-dependent transcription [23, 24] and to reduce MYC protein stability [25]. In addition, JQ1 has previously been demonstrated to inhibit multiple members of the AP-1 TF complex by reducing both total JUN expression and JUN phosphorylation [26–29]. We identified and re-analyzed 3 publicly available datasets in which SMC or fibroblasts were treated without or with JQ1: rat vascular SMCs exposed to PDGF (GSE111714); human vascular SMC stimulated with undefined growth medium (GSE138323); and rat cardiac fibroblasts stimulated TGFβ1 (GSE127229) [30]. PCA plots of the count and expression matrices of the publicly available data were visualized to determine quality of replicates and separation of conditions (Supplementary Fig. 8A-C). Greater than 90% of the variation for each dataset was accounted for by the first two principal components demonstrating that most of the differences were due to experimental stimulations. DEGs were generated using DESeq2 and filtered as mentioned in the methods followed by pattern analysis using the degPatterns function from the DEGreport package [31]. This analysis identified two groups of genes from each dataset: those that were either up- or down-regulated with the stimulus, and subsequently attenuated with JQ1 co-treatment (Fig. 2A, C, and E). Genes that fit this pattern were classified as JQ1-sensitive genes.

Enrichment analyses on JQ1-sensitive DEGs was performed using GO-CC terms (Fig. 2B, D, and F) as well as GO-MF terms (Supplementary Fig. 9A-C). We combined the two sets of JQ1-sensitive DEGs identified in each dataset for this analysis. In rat VSMCs treated with PDGF (GSE111714), JQ1 attenuated changes related to chromatin remodeling (cluster 6, fold enrichment = 1.78, *p*-value = 9.95e-10) and the cytoskeleton (cluster 13, fold enrichment = 2.04, *p*-value = 7.77e-03) (Fig. 2B). Similarly, in human VSMC stimulated with growth media (GSE138323), JQ1 attenuated changes corresponding to chromatin remodeling (cluster 7, fold change = 2.50, *p*-value = 2.30e-09 and cluster 13, fold change = 1.41, *p*-value = 1.27e-07). In cluster 39 (data not shown), genes involved in the actin cytoskeleton were enriched (fold change = 1.28, *p*-value = 8.91e-04) (Fig. 2D). Notably, the growth media stimulation did not result in the identification of a group of genes that were down regulated compared to control, and subsequently returned to near base line expression with JQ1. Although the datasets describe two different treatments in VSMC from two species, JQ1 was able to attenuate expression of genes associated with chromatin remodeling and the actin cytoskeleton.

Transcriptomic dataset GSE127229 was generated from rat cardiac fibroblasts treated with TGFβ1 ± JQ1 and analyzed as previously mentioned. Two groups of JQ1-sensitive genes were identified in which TGFβ treatment perturbed gene expression compared to vehicle (DMSO)-treated control and were attenuated by JQ1 (Fig. 2F). These groups of JQ1-sensitive genes were combined for each dataset to perform enrichment analysis for GO-CC and GO-MF terms. Both chromatin and cytoskeleton-related changes were enriched in the JQ1-sensitive genes in this fibroblast dataset. Notably, the cytoskeleton-associated processes were enriched to a greater extent in the fibroblast dataset compared to the SMC datasets. Additionally, these cytoskeleton-associated terms were more highly enriched than chromatin-associated terms within each dataset compared. Overall, these JQ1-sensitive, cytoskeleton-associated genes from SMCs and fibroblasts were well represented in the KEGG pathway, “Regulation of the actin cytoskeleton”, and JQ1 produced > 20% reduction in the most robustly expressed genes (Supplementary Fig. 10A, B). This further supports the hypothesis for a novel mechanism whereby treatment with JQ1 can regulate effectors of the cytoskeleton not only within fibroblasts, but also in SMC albeit to a lesser extent.

### Identifying BRD4-dependent and -independent JQ1 sensitive cytoskeleton associated genes

To refine our list of genes for in vitro validation, we sought to identify which genes were dependent or independent of BRD4 inhibition via JQ1. To understand this, an additional RNA-seq dataset comprising IMR90 human airway fibroblasts, under conditions of quiescence or growth and treated with DMSO, JQ1, control shRNA, or shBRD4 (GSE74324)[32] was analyzed. Nearly 75% of the total variation in the dataset was attributed to the difference between the conditions (Supplementary Fig. 11A, B). Pattern analysis was performed to identify JQ1-sensitive and BRD4-sensitive DEGs, defined as those that were perturbed by growth conditions and returned to near baseline levels with JQ1 and or knock-down of BRD4 (BDR4-KD) (Supplementary Fig. 12). Genes that were attenuated by both JQ1 and BRD4-KD were classified as BRD4-dependent. Genes uniquely sensitive to JQ1 were considered as BRD4-independent. The third group of genes were classified as BRD4 unique (Fig. 3A). These 3 sets of genes were subjected to TF enrichment analysis using ChEA3 as described earlier. MYC and members of the AP-1 TF complex were enriched in all three gene sets (Fig. 3B). This supports a mechanism for JQ1-mediated modulation of a MYC-regulated network of genes in both a BRD4-dependent and a BRD4-independent manner. This is not surprising as JQ1 has been shown to inhibit MYC-regulated genes through targeting additional BET family members such as BRD2 [33]. Of 21 transcriptional regulators assessed, including the top enriched TFs identified in the ChEA3 analysis, only 3 were found to be regulated transcriptionally by JQ1 and not BRD4-KD (Fig. 3C). These included FOSL2, RUNX1, and EGR1, previously shown to be sensitive to pro-fibrotic signals [8]. Further, JQ1 robustly and uniquely induced the expression of BRD2, supporting previous studies of JQ1 modulation of BRD2 activity [33]. Taken together, these data imply that JQ1 regulates MYC post-transcriptionally to modulate a subset of genes in its network.

Following TF enrichment analysis, the up- and down-regulated JQ1- and shBRD4-sensitive DEGs were stratified for GO-term enrichment analysis. JQ1- and BRD4-KD down-regulated genes shared GO-CC terms for cytoskeleton, stress fiber, focal adhesion, and actin cytoskeleton (all showing statistically significant fold enrichment > 1.85) (Fig. 3D). There were no terms related to the cytoskeleton that were sensitive to JQ1 only, i.e. uniquely enriched in the up- or down-regulated, JQ1-responsive DEGs (Supplementary Fig. 13A, B). This suggests that the regulation of cytoskeleton-related changes is not exclusively a result of BRD4-independent effects of JQ1.

The evidence for regulation of cytoskeleton-related terms shared between BRD4-KD or JQ1 treated cells supports a BRD4-dependent mechanism. To examine this at the gene level, five gene lists were compared via an upset plot (Supplementary Fig. 14A) to determine which JQ1-sensitive cytoskeletal genes from the previous analyses (Supplementary Fig. 10B) were BRD4-dependent or -independent. Four groups comprising 30 genes were visualized using two KEGG pathways that best represent the processes in which they are enriched: ‘Regulation of the Actin Cytoskeleton’ and ‘Focal adhesion’ (Supplementary Fig. 14B, C). A representative gene from each of the four groups was identified for in vitro validation based on their overlap with the most robustly expressed genes from Supplementary Fig. 10B. These genes included ITGB1 (The only gene in Group 1), ACTN1 (Group 2), CFL1 (Group 3), and ITGA5 (Group 4). Additionally, we selected 3 kinases not predicted to be attenuated by JQ1, but that are well established as an initiator (PDGFRA), propagator (SRC) and facilitator (LIMK1) of cytoskeletal changes in mechanically active cells [34, 35].

### In vitro validation of JQ1-sensitive cytoskeletal targets

To validate the predictions from the in silico analyses we assessed the sensitivity of the subset of cytoskeleton-associated genes to JQ1 in rat bladder mesenchymal cells (RBMC) and human bladder SMC (pHBSMC). RBMC were identified as fibroblasts due to higher relative expression of fibroblast marker Mfap5 and lower relative expression of SMC markers including Cnn1, SM22α, and αSMA compared to pHBSMC (Supplementary Fig. 15A). An optimal dose of 0.5 µM JQ1 was determined using crystal violet growth assay (Supplementary Fig. 15B). Cells were then stimulated with PDGF or TGFβ with or without JQ1 for up to 16 h, and expression of 8 genes was assessed via PCR: Platelet derived growth factor receptor alpha (Pdgfrα), Integrin beta 1 (Itgb1), Integrin alpha 5 (Itga5), actinin 1 (Actn1), Src, non-receptor tyrosine kinase (Src), Beta actin (Actb), Lim kinase (Limk) and cofilin (Cfl1). Notably, PDGF and TGFβ induced expression of all 8 targets at 2 h in RBMC (1.34, 1.43, 0.97, 1.43, 1.60, 1.01, 0.60, 1.00 and 0.63, 1.05, 1.02, 1.28, 1.68, 1.13, 0.83, 0.88 log2FC respectively)(Fig. 4A). All targets except ActB and Actn1 were suppressed to near baseline levels by JQ1 as early as 2 h post stimulation with PDGF or TGFβ. By 16 h, JQ1 suppressed PDGF and TGFβ induction of only Pdgfrα, Itga5, Src and Actn1. pHBSMC showed similar reduction in expression of all target genes with JQ1 by 16 h regardless of PDGF or TGFβ stimulation (Supplementary Fig. 16). Overall, the validation of JQ1 sensitivity of a subset of cytoskeletal effectors suggests the use of JQ1 to modulate MYC-regulated cytoskeletal changes.

We also determined which targets were sensitive to JQ1 at the protein level (Fig. 4B, C). Notably, PDGFRα, Src, Itga5 and Itgb1 decrease with JQ1 regardless of PDGF or TGFβ stimulation at 16 h. Additionally, we observed a reduction in the transcriptional expression of contractile proteins such as Myh11, SM22α and αSMA in both pHBSMCs and RBMCs (Supplementary Fig. 17A). Additionally, Cnn1, but not SM22α was found to be reduced at the protein level (Supplementary Fig. 17B). This further suggest that the contractility of both cell types may be sensitive to JQ1.

### JQ1 alters the cytoskeleton and inhibits contraction in SMC and fibroblasts

**Fig. 6:**
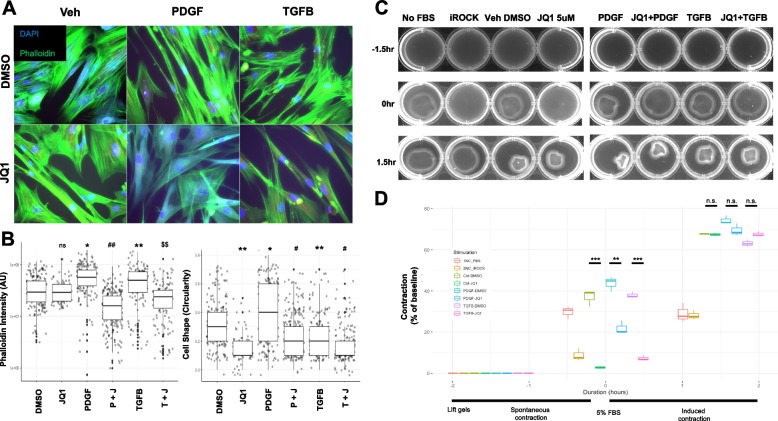
In vitro functional validation of JQ1 attenuation of PDGF and TGFB stimulated contraction in pHBSMC. **A** Representative images of pHBSMC stimulated with PBS (Veh), PDGF, TGFB with DMSO or JQ1 for 16 h and stained with Vimentin, Phalloidin and DAPI. **B** Phalloidin intensity and cell shape was quantified in ImageJ. **C** RBMC were plated on 1.2 mg/mL collagen gels in a 12well plate in 1 mL of serum free media and incubated for 16 h with PBS (Veh), PDGF, TGFB with DMSO or JQ1. Select wells were stimulated for 30 min with a ROCK inhibitor as a negative control for contraction. Collage gels were separated from the walls of the wells and Imaged after 1.5 h to capture spontaneous (unstimulated contraction, followed by an additional 1.5 h in 5% FBS to facilitate contraction. **D** Contraction was quantified using ImageJ and measured as a percent of the changed area of the gel from the baseline. Data are representative of 3 independent trials. 1NC_FBS refers to negative control for FBS in which cells never receive FBS from platting to harvesting. 2NC_iROCK refers to the negative control for contraction in which cells are stimulated with a ROCK inhibitor for 30 min prior to observing contraction. *, *p* < 0.05, ** *p* < 0.01, ***, *p* < 0.001, ****, *p* < 0.0001 compared to control. #, *p* < 0.05, ## *p* < 0.01, ###, *p* < 0.001, ###, *p* < 0.0001 compared to PDGF. $, *p* < 0.05, $$ *p* < 0.01, $$$, *p* < 0.001, $$$$, *p* < 0.0001 compared to TGFB. Statistical significance was calculated with student ttest

To determine whether the changes in expression of cytoskeleton-associated genes and proteins observed with JQ1 treatment altered cell behavior, we determine how JQ1 impacts cytoskeletal and contractile changes induced by PDGF and TGFβ. Both human and rat cells were assessed using fluorescence-based imaging of phalloidin to measure changes to the actin cytoskeleton (Fig. 5A and 6A). PDGF and TGFβ altered the cytoskeleton of both human and rat cell lines compared to baseline. PDGF induced a collapse-like phenotype whereas TGFβ increased actin fiber formation as reported previously by our group and others [7, 36–38], with JQ1 preventing these changes in both cell types. The most notable difference in both cell types was observed between TGFβ-treated cells in the absence and presence of JQ1, with RBMC exposed to JQ1 + TGFβ showing > sixfold reduction in phalloidin intensity compared to RBMC treated with TGFβ1 alone (0.89e07 AU for TGFβ + JQ1 vs 6.02e07 (AU) for TGFβ) (Fig. 5B). In pHBSMC JQ1 inhibited a 20% increase in phalloidin intensity induced by TGFβ compared to DMSO control (1.76e07 AU for TGFβ vs 1.43e07 AU for TGFβ + JQ1) (Fig. 6B). Another difference between the two cell types in response to TGFβ included cell shape changes. Here, cell shape is defined as the circularity of the cell, defined further in the methods. TGFβ stimulation of RBMC resulted in a threefold increase in the circularity compared to control DMSO + Veh. JQ1 inhibited this TGFβ stimulated shape change. Interestingly, TGFβ stimulated pHBSMC showed a 50% decrease in circularity compared to control. This decrease in circularity was enhanced by JQ1 + TGFβ combination treatment compared to the DMSO control (0.2 to 0.1 respectively).

To determine if the changes we observed in the cytoskeleton that were attenuated by JQ1 had functional consequences we utilized an optimized and modified collagen gel contractility assay detailed in the methods. Contraction was assessed in the context of concurrent PDGF, TGFβ or vehicle (Veh) stimulation with JQ1 (5 µM) or DMSO for 16 h. These parameters were determined in preliminary analysis as described in Methods. At baseline (Veh DMSO), both RBMC and HBSMC contracted collagen gels during both the spontaneous and elicited contraction phases (Figs. 5C, D and 6C, D). Spontaneous contraction was inhibited with the ROCK inhibitor Y-27632 (iROCK, 10 µM) in both cell types, albeit to a lesser extent in pHBSMC. Stimulation with PDGF and TGFβ for 16 h resulted in increased contraction above baseline for RBMC in the elicited contraction phase, which was attenuated with JQ1 pretreatment. In contrast, the magnitude of contraction by pHBSMC in the elicited contraction phase was not different whether cells were treated with PDGF, TGFβ or JQ1. To rule out the possibility that JQ1-mediated inhibition of contraction resulted from cytotoxicity, cell viability was assessed in a dose- and time-dependent manner using Alamar Blue (Supplementary Fig. 18A, B). This analysis showed that JQ1 did not affect viability at doses up to 20 µM following treatment for 16 h.

### Inhibition of MYC dimerization destabilizes the cytoskeleton and reduces contractility similar to JQ1 in RBMC

**Fig. 7:**
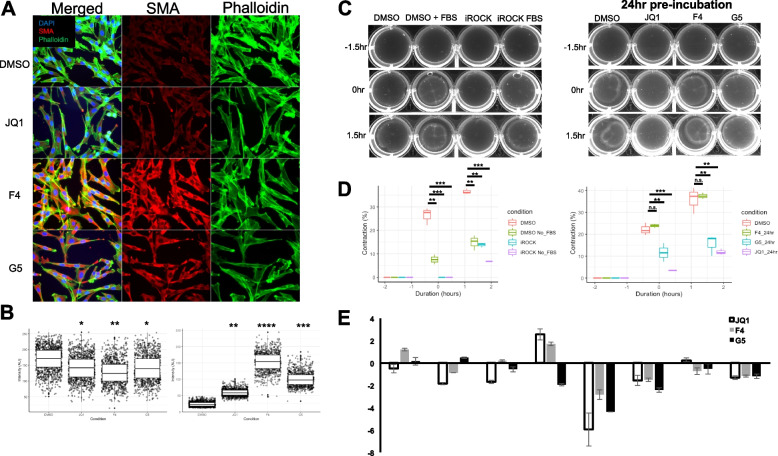
Inhibition of MYC–MAX dimerization destabilizes the cytoskeleton and reduces contractility similar to JQ1. RBMC were plated in 4 well chamber slides and incubated with DMSO, JQ1, MYC-MAX dimerization inhibitors 10,048-F4 (F4), or 10,074-G5 (G5) for 16 h. Cells were stained for αSMA, Phalloidin or DAPI. Forty images were taken and stitched together with ImageJ to make a 5 × 8 field of view. **B** Phalloidin and αSMA intensity were quantified for each cell using ImageJ and plotted for each condition. **C** Cells were plated on collagen gels and stimulated for 16 h with DMSO, JQ1, F4 or G5 in serum free media and spontaneous contraction of gels was captured for 1.5 h prior to adding FBS to a final concentration of 5% to elicit contraction. **D** Contraction was quantified as a percentage in the change in area of the gel compared to baseline. **E** RNA was harvested from cells stimulated with DMSO, JQ1, F4 and G5 for 16 h and predicted JQ1 sensitive cytoskeleton associated genes was measured with qPCR

JQ1 inhibits MYC indirectly through targeting of the bromodomain-containing proteins BRD4 and BRD2 [39]. To further explore the impact of MYC inhibition on the cytoskeleton and contractile phenotype, we tested 2 additional MYC inhibitors with a distinct mechanism of action, namely the MYC-MAX dimerization inhibitors 10,048-F4 (F4) and 10,057-G5 (G5). Cytoskeletal changes were assessed using phalloidin staining. Cells were co-stained with DAPI and αSMA, as a marker of fibroblast activation (Fig. 7A). Phalloidin and αSMA intensity were quantified using ImageJ (Fig. 7B). All three MYC inhibitors reduced phalloidin intensity by more than 20%. αSMA signal, however, was increased by all inhibitors with the largest increases induced by F4 and G5 by sixfold and fourfold respectively. This different response likely reflects the direct and indirect mechanism of MYC inhibition by dimerization inhibitors versus JQ1.

In the collagen gel contraction assay, JQ1 and G5, but not F4 were able to inhibit contraction from 21.1% in the DMSO control to only 4.6% and 11.4% during the spontaneous contraction phase, respectively. JQ1 and G5 were also able to inhibit elicited contraction from 37.2% with DMSO to 10.8% and 18.1% respectively. (Fig. 7C, D). The inhibition of RBMC contraction was coupled with reduced expression of cytoskeleton-associated genes as determined by qRT-PCR. All three inhibitors decreased expression of Pdgfrα, Itgb1 and Actn1 (Fig. 7E). Interestingly, there was no consensus on shared changes in smooth muscle contractile gene expression. However, JQ1 consistently down regulated αSMA, Cnn1, and Cald1.

## Discussion

In this study, we build upon our prior demonstration of a MYC-centric network in SMC that regulates proliferation, to show that inhibition of MYC also attenuates contraction. We show that (i) PDGF- and TGFβ1-induced signals converge on MYC/MAX in smooth muscle cells and fibroblasts; (ii) JQ1 treatment attenuates the expression of cytoskeleton-associated genes; and (iii) inhibition of MYC with two independent inhibitors reduces cytoskeletal stability and contraction of mechanically active cells. Together, these findings suggest that JQ1 could be used to moderate aberrant contraction in addition to pathological fibroproliferative remodeling in hollow organs.

Although the ability of JQ1 to target MYC activity has been most widely studied in the context of cancer [40], a number of recent studies have investigated the utility of JQ1 in non-cancer settings including contractile tissues. Lim and colleagues evaluated the ability of JQ1 to inhibit myometrial cell contraction in vitro and in vivo. In that study, either JQ1 treatment or knockdown of BRD2/3/4 were found to inhibit TNFα-stimulated adhesion and contraction of myometrial cells. JQ1 also inhibited inflammation-induced premature labor contractions in mice [41]. Notably, however, JQ1 was found to elicit its effects through inhibition of NFkB transcriptional regulation. Importantly the role of MYC was not assessed in that study. As a result we sought out and reanalyzed an additional dataset in which Rheumatoid Synovial fibroblasts were stimulated with the proinflammatory cytokine IL1β (GSE148395) (Supplementary Table 2) [42]. We found similar inhibition of IL1β-stimulated cytoskeleton-associated genes with JQ1 as with PDGF, TGFβ and growth media. The JQ1-sensitive genes identified in that dataset, of which a subset were associated with the cytoskeleton, were predicted to be largely regulated by MYC/MAX and AP-1 TF complex members (data not shown). These findings suggest that targeting MYC and AP-1 may also attenuate changes associated with the cytoskeleton and contraction induced by pro-inflammatory stimuli. Similarly, Duan et al. showed that JQ1 treatment could attenuate deleterious functional changes, including reduced left ventricle ejection fraction, in mouse models of cardiac injury [43], also through attenuation of NFkB signaling networks. In contrast, Yan and colleagues showed that JQ1 could inhibit contractile responses in mouse aorta, rapidly and at high doses [44]. Moreover, a similar effect was observed with both ( +)-JQ1 and its enantiomer (-)-JQ1 at 100 µM, the latter of which cannot inhibit BRD4 recognition of acetylated histones [23]. These observations together with the demonstration that the inhibitory effect of ( +)-JQ1 on smooth muscle contraction could not be replicated with knockout of BRD4, suggested that the observed effect of JQ1 in that study reflected off-target activity. In contrast to Yan et al. short incubations with JQ1 in our analyses did not affect spontaneous or elicited contraction. Moreover, in our study cytotoxicity was observed at fivefold lower concentrations (20 µM) than those used by Yan and colleagues. Therefore our studies support a mechanism for aberrant contractile inhibition by JQ1 through the repression of the MYC and likely Jun TF regulator gene networks.

Previous studies have established a connection between MYC and contraction through interactions with an oncogenic form of RHOA. However, this relationship has been shown to down regulate the F-actin stress fiber formation [13, 15]. Additionally, overexpression of MYC alone has been shown to be sufficient to destabilize the actin cytoskeleton [17]. Notably, these studies attempted to recapitulate a cancer like state though over expressing MYC, which does not reflect the physiologically relevant stimulation of MYC. Our data demonstrate that MYC inhibition destabilizes the actin cytoskeleton. Taken together, the JQ1 mediated inhibition of physiologically relevant stimulation of MYC that we have demonstrated combined with MYC overexpression studies supports a complex role for MYC expression dynamically influencing cytoskeletal rearrangement and stabilization in mechanically active cells in addition to its well-studied oncogenic effects in other cell and tissue types [45–48].

Prior findings from us and others have demonstrated opposing effects of PDGF and TGFβ on SMC, with the former acting as a canonical mitogen and the latter, a potent growth inhibitor [21, 49]. Interestingly, however, in silico analysis of publicly available data revealed significant overlap in the genes differentially regulated by PDGF and TGFβ in both SMC and fibroblasts, with more than 80 DEGs regulated by MYC, MAX, and JUN in master regulator analysis. Comparative analysis between our previously published data of PDGF stimulated pHBSMC showed nearly 30% similarity with datasets of fibroblasts and smooth muscle cells stimulated with PDGF or TGFβ1. These observations agree with data from Ghosh et al., which established that PDGF and TGFβ shared significant overlap of differentially expressed genes within mesenchymal stem cells [50]. In that study, the authors proceeded to compare cells stimulated with each cytokine alone to those exposed to both concurrently, adding that the combination influenced cell stiffness through synergistic induction of integrin expression. Notably, the genes encoding α5 and β1 integrins (Itga5 and Itgb1) were induced in the datasets we analyzed and attenuated with JQ1 in silico and in vitro. Consistent with their role in anchoring cells to their substrate, the reduction in or loss of mediators of cell-ECM connections, such as integrins, has been shown to alter contraction [51, 52]. Our data support a model in which JQ1 reduces aberrant contraction, at least in part through repressing MYC-mediated regulation of Itga5 and Itgb1.

It has been shown that aberrant contraction is a consequence of alterations to the cytoskeleton of mechanically active cells [2, 53]. We show that JQ1-induced destabilization of the cytoskeleton results in reduced contraction. Interestingly, These findings agree with those of Stratton et al. who showed that JQ1 reduced baseline and TGFβ1-stimulated cytoskeletal stabilization of cardiac fibroblasts, with a corresponding decrease in collagen gel contraction [30]. In that study the authors did not address the role of MYC. Instead NFkB was found to mediate this process. In our study, we observed that the effect of JQ1 could be replicated with another MYC inhibitor with a different mechanism of action. Similarly, Wang and colleagues identified that JQ1 and the MYC inhibitor 10,048-F4 repressed MYC expression in rat and rabbit lens epithelial cells [54]. This resulted in reduced EMT as manifested by αSMA and fibronectin expression further supporting a MYC dependent phenomenon of JQ1 inhibition. Our data support that the inhibitory effect of JQ1 on aberrant contraction is being mediated through inhibiting MYC in mechanically active cells.

While this study provides evidence of a MYC centric network of aberrant gene expression in smooth muscle cells and fibroblasts stimulated by PDGF and TGFβ, these comparisons have been limited to in silico and in vitro analyses. As mentioned previously, there have been several studies that have utilized JQ1 to attenuate pathological changes in various animal models of organ injury [20]. However, most have not implicated or compared MYC activity and inhibition in the mechanism of action for JQ1 in the attenuation of those changes. Our data support a potential for repurposing the anti-cancer, therapeutic JQ1 to inhibit MYC in a non-cancer setting. However, understanding the extent to which MYC expression and activity can be different in these contexts will provide further rationale for its efficacy. Finally, when considering the primary target of JQ1 activity, BRD4, which prevents MYC recruitment and transcriptional activity, there are other BRD proteins that are regulated by JQ1 [33]. We have reanalyzed RNA-seq dataset GSE74324 in which human airway fibroblasts were treated with JQ1 or BRD4-KD under conditions of growth or quiescence. These data support a mechanism by which JQ1 not only inhibits BRD4, but also regulates other BET family members transcriptionally. Further, cytoskeleton-associated terms shared exclusively between JQ1-treated and BRD4-KD cells supports a mechanism whereby JQ1 facilitates cytoskeleton-related changes in a BRD4 dependent manner.

## Conclusion

Utilizing in silico and in vitro techniques we determined that the inhibitor, JQ1, attenuates MYC-driven, aberrant cytoskeletal changes and contraction induced by PDGF and TGFβ1 treatment of smooth muscle cells and fibroblasts. Our findings support a shared, MYC-regulated, gene network stimulated by PDGF and TGFβ1 in mechanically active cells. We showed that JQ1 attenuates aberrant contraction and cytoskeletal changes through regulating the expression of various cytoskeleton associated genes. Further, comparing the effects of MYC-MAX dimerization inhibitors with JQ1 suggest that there are partially overlapping gene targets between BRD4 and MYC-MAX transcription factors that result in similar impacts to the cytoskeleton and contraction.

## Methods

### In silico analyses of publicly available microarray data

We re-analyzed gene expression data generated previously by us [8] and compared the results with re-analyzed publicly available data of SMC and fibroblasts stimulated with PDGF and TGFβ1. Data were retrieved from the NCBI GEO Database using GEOQuery [55]. Four datasets were retrieved; GSE52488 (SMC stimulated with PDGF), GSE63383 (SMC stimulated with TGFβ), GSE61128 (Fibroblasts stimulated with TGFβ), and GSE14256 (SMC stimulated with PDGF). Data quality was assessed using PCA plots to determine how replicates of various stimulations clustered together using the base R stats package [56]. Well-clustered replicates that were separated from the other stimulations within a dataset were analyzed using linear models for microarray analysis (LIMMA) packages in R [57]. Two approaches were used to perform enrichment analysis. The first approach utilized a traditional GSEA method. Roughly 800 gene for each gene sets were rank ordered based on Log2FC. A running-sum statistic was performed in which the score increased or decreased with the presence or absence of a gene from in the rank order list in the GO term gene list, respectively. The second approach subjected the DEGs from each comparison as well as Log2 fold changes and *p*-values into the pathfindR package in R [58]. This tool performs active subnetwork searches against the BIOGRID database of protein–protein interaction networks. All genes in the most significant subnetworks are subjected to the enrichment analysis. Separate analyses were run using GO terms for molecular function, biological process and for cellular compartment.

### In silico analyses of publicly available RNA-seq data

We reanalyzed publicly available data which included rat VSMCs exposed to PDGF (GSE111714), human VSMC stimulated with undefined growth medium (GSE138323), rat cardiac fibroblasts stimulated TGFβ (GSE127229) [30], Rheumatoid Synovial Fibroblasts stimulated with IL1B (GSE148395) [42] and IMR90 human airway fibroblasts treated without or with JQ1, or in which BRD4 was knocked down using RNAi (GSE74324) [32]. All datasets contained conditions of JQ1 with and without the respective stimulus. For GSE111714, GSE148395, and GSE127229, SRA raw files were downloaded from the SRA run selector using the SRAtoolKit [59], genome indexes were generated using the most recent versions of the appropriate species primary assemblies, aligned to the appropriate species library using STAR aligner [60], count matrices were generated using the Subreads, FeatureCount function [61] and the count matrices were analyzed with DESeq2 [62]. Data frame manipulations were performed using a variety of packages in R including tidyR [63], dplyR [64], tibble [65], and stringR [66]. Plots were constructed using ggplot2 [67] and GGally [68]. Conversion of Ensembl and Affymetrix microarray gene IDs was performed using a combination of biomaRt [69], and Hs.eg.db [70]. Venn diagrams of overlapping genes were generated with ggVennDiagram [71] and Volcano plots were generated using EnhancedVolcano [72]. Pattern analysis of DEGs was performed using the DEGpattern [31] package. ClusterProfileR [73] and pathfindR [58] were utilize to perform enrichment analysis using GO terms for molecular function, biological process and cellular compartment on DEG lists without and with Log2FC and adjusted *p*-values, respectively. Hierarchical clustering of enriched terms was performed to cluster similar groups of terms together. Briefly, the clustering algorithm in the cluster_enriched_terms function uses a method that calculates the maximum number of clusters to generate based on the number of enriched terms and the extent of overlap of genes in common across each term using 1—k as the distance metric to compare enriched term proximity and therefore grouping into the same or separate clusters.

### ChEA3 Transcription factor enrichment analysis

The ChEA3 transcription factor (TF) enrichment analysis tool [22] was used to identify and rank TFs that regulate the list of DEGs from each comparison. Lists of shared DEGs from each comparison were submitted to the ChEA3 online tool. Enrichment of TFs that regulate the DEGs was determined using the ENCODE ChIP-seq library as the reference for TF-target gene interactions. The gene list is then compared to the annotated ENCODE ChIP-seq library to determine enrichment of TFs for groups of genes from the gene list. The enriched TFs are then rank ordered based on Fisher’s exact test. Bar charts showing the -log Fisher's exact test (FET) *p*-value were generated for the top ten TFs.

### Cell culture

Primary human bladder smooth muscle cells were purchased from ScienCell (Cat. No. 4310, Lot# 6585) and cultured in Smooth Muscle Cell Medium (ScienCell, Cat. No. 1101) with Penicillin/streptomycin (Cat. No. 0503), 2% FBS (Cat. No. 0010) and smooth muscle cell growth supplement (Cat. No. 1152). Cells were used up to passage 6. Rat bladder mesenchymal cells were isolated from P10 rat pups as follows: 20 bladders were minced in 1 ml of 1 × PBS post dissection and incubated for 1 h in a dissociation solution containing: 1.25 mg/ml Elastase, type III 1 (E0127, 20 mg, Sigma), 1 mg/ml Collagenase I (C0130, 1 g, Sigma), 0.25 mg/mL Trypsin Inhibitor (soybean, T9003, 100 mg, Sigma), BSA, 2 mg, and 0.2 mL of pen/strep (100 X) in 20 mL of M199 media (11,150,059, 500 mL Thermo Fisher). Following incubation, the dissociated cell suspension was filtered through a 100 µm filter, centrifuged at 176 × g for 3 min, resuspended in 10 mL of M199 containing 20% FBS and plated in a 10 cm dish. Cultures were passaged 4 times before cells spontaneously immortalized. RBMC were used at passages 16–20.

### Validation of in silico predicted JQ1 sensitive genes using qRT-PCR

All JQ1 sensitive genes from both the PDGF and TGFβ datasets were filtered for genes identified in the KEGG pathway, “Regulation of the Actin Cytoskeleton”. A total of 39 unique genes were identified between the two datasets and visually represented in the pathway. Two additional criteria were imposed for consideration of in vitro validation. These included a minimum baseline expression 3000 counts or greater and a difference in expression of 20% or greater between the co-treatment condition compared to the perturbation alone. 16 genes remained after applying these filters. Finally, these genes were filtered based on whether they overlapped with 1 of four groups of genes identified in Supplementary Fig. 10B. This left 4 genes, each representing 1 of the four groups (ITGB1, ACTN1, CFL1, ITGA5). 3 Additional targets were chosen as well established, representative initiator (PDGFRA), propagator (SRC) and facilitator (LIMK1) of cytoskeletal changes. Primers to all seven targets were designed for human and rat transcripts using NCBI primer design tool such that the length of the product was between 100–300 bases to facilitate rapid detection via qPCR and ordered from Integrated DNA technologies. RBMC and pHBSMC were plated in 6 cm dishes at 70% confluence in complete media (DMEM, 10% FBS, pen/strep). After an overnight incubation, the media was changed to low serum media (DMEM, 0.5% FBS, pen/strep) (Thermo Fisher) for 24 h. Cells were then stimulated with DMSO (Sigma Millipore), PBS (Thermo Fisher), JQ1 (5 µM) (Cayman Biochemical), PDGF (2.5 ng/mL) (R&D systems) or, TGFβ1 (2.5 ng/mL) (R&D systems). Cells were harvested after 24 h using 500µL of TRIzol. 100µL of chloroform was added to the Trizol, mixed by vortexing and centrifuged for 15 min at 7826 × g. The aqueous phase was separated and added to an equal volume of 70% ethanol before using the RNeasy minikit (Qiagen) to isolate RNA following the manufacturer’s protocol. cDNA was generated using the iScript cDNA synthesis kit (Bio-Rad) following the manufacturer’s protocol. qRT-PCR was performed using 18 ng of sample cDNA with 1uL of premixed primers and 10uL of 2 × SYBR select master mix (Thermo Fisher) and run in a QuantStudio3 thermocycler (Thermo Fisher). Expression of each target was normalized to Gapdh, Rps18 as housekeeping genes with the least variation in cycle values across all samples. Notably, ACTB could not be used as a house keeping gene as the standard deviation across samples was greater than 1 cycle and had a range of 4.78 cycles, while GAPDH and Rps18 had standard deviations of 0.742, and 0.148 respectively and a range in cycle values of 3.04 and 0.59 respectively (data not shown).

### Validation of in silico predicted JQ1 sensitive genes via immunoblots

Targets assessed via immunoblot included PDGFRβ, PDGFRα, ITGB1, ITGA5, αACTN, SRC, and CFL (Cell Signaling Technology). Additional targets assessed included MYC, SM22α (Cell Signaling Technology), CNN1, Vim, αSMA and ACTB (Sigma). To assess these targets via immunoblot, cells were stimulated as before in 10 cm dishes, washed with 1 × PBS and then lysed on ice using 1 × cell lysis buffer (Cell Signaling Technology) containing SDS. The lysate was mixed with 4 × sample buffer (1% Bromophenol blue, 50% glycerol, 0.125 M Tris–HCl, pH 6.8, 4% SDS) and boiled for 15 min. Lysates were resolved on 10% and 15% polyacrylamide mini gels at 100 V for 20 min then 160 V for 70 min. Gels were transferred to nitrocellulose membranes at 100 V for 2 h at 4 °C. Nitrocellulose membranes were stained with Ponceau S to confirm transfer of total protein then washed with distilled water for 5 min, PBS-T for 5 min and blocked in 10% milk for 1 h. Membranes were then washed briefly with PBS-T and incubated in one of the aforementioned primary antibodies overnight at 4 °C. Membranes were then washed 3 times for 15 min in PBS-T prior to being incubated in 2° HRP conjugated anti-rabbit or anti-mouse antibody for 1 h at room temperature in 10% non-fat powdered milk followed by 3 washes in PBS-T. The washed membranes were then subjected to 5 min incubation with SuperSignal™ West Pico PLUS Chemiluminescent Substrate (Thermo Fisher) following the manufacturers protocol. Signal was captured by ChemiDoc chemiluminescence imaging or on HyBlot CL™ Autoradiography Film (Thomas Scientific) after 10 s, 1 min, 1 h, 1 min, 30 s, 10 s exposures. Films were digitized using an Epson scanner and quantified with FIJI.

### Assessment of cytoskeleton changes

pHBSMC and RBMC were plated into 2 × 4 well chamber slides (Thermo Fisher) at a concentration of 50 k cells/well in complete medium. Following an 8 h incubation at 37 °C and 5% CO_2_ the media was changed to DMEM with 0.5% FBS and incubated for 24 h. Cells were stimulated with DMSO, PBS, JQ1, PDGF, TGFβ1, JQ1 + PDGF, JQ1 + TGFβ1 for 24 h followed by fixation using 4% paraformaldehyde for 15 min at room temperature. Post fixation, cells were blocked and permeabilized using blocking buffer (5% FBS, 0.1% BSA, 3% Triton X in PBS) for 1 h at room temperature. Permeabilized cells were then incubated overnight in Alexa Fluor 488 phalloidin (Thermo Fisher), anti-αSMA, anti-MYC. The cells were washed in 1 × PBS 3 times for 5 min and then incubated in incubation buffer (1% FBS, 0.1% BSA in PBS) for 1 h containing species specific 2° Antibodies conjugated to Alexa Fluor 594 or Alexa Fluor 647 (ThermoFisher). The cells were then washed again, and a cover slip was added after addition of DAPI containing mounting medium (Vector Labs). The cells were imaged using a Zeiss fluorescence scope. 20–30 images were taken for each condition to ensure that more than 100 cells were captured per condition. Fluorescent signal and cell shape was quantified via FIJI based macro. The circularity of the cell is calculated as the ratio of the second longest dimension of the cell that runs perpendicular to the longest dimension of the cell. The closer the ratio is to 1 the more rectangular the cells. Long thin cells will have a circularity closer to 0.

### Assessment of changes in contractility

Contractility was assessed using a modified collagen gel contractility protocol. Briefly, Collagen gels were made using 2.4 mL 10 × PBS, 157uL of 1N NaOH, 14.07 mL of ddH_2_O, 6.56 mL of rat tail type 1 collagen (Advanced Biomatrix), all reagents were kept on ice until ready to mix. Once mixed, 0.75µL of the solution was added to every well of 2 × 12 well plates. Gels were incubated at 37 °C in 5% CO_2_ for 1 h to polymerize. Cells were then lifted, counted, spun down to remove trypsin containing media, and resuspended in DMEM with 0.5% FBS and Pen/Strep at a concentration of 200 k cells/mL. 1 mL of cell suspension was added to each well containing a solidified collagen gel. Cells were incubated overnight at 37 °C in 5% CO_2_. Cells on gels were then stimulated for 16 h with DMSO, ROCK inhibitor Y-27632 (iROCK), PDGF, TGFβ1, JQ1, PDGF + JQ1, or TGFβ1 + JQ1. Gels were then separated from walls and bottom of the well and imaged every 30 min for 1.5 h using a chemi-doc imaging platform (Bio-Rad). This first 1.5 h was considered the spontaneous contraction phase (SCP). After the SCP, 100uL of FBS was added to each well containing cells on gels to initiate the elicited contraction phase (ECP). 16 h pretreatment with 0.5 µM JQ1 led to modest inhibition of contraction, while 5.0 µM JQ1 lead to significantly inhibited contraction with limited reduction in viability. All doses of JQ1 at 24 h reduced viability by nearly 30%. As a result, 5 µM JQ1 pretreatment for 16 h was used for all additional collagen gel contraction assays unless otherwise noted Images were taken every 30 min for 1.5 h and captured with the Chemi-doc imaging platform. Images were analyzed in FIJI and the data was plotted in R using ggplot2.

### Supplementary Information

**Additional file 1.**

**Additional file 2:** **Supplementary Figure 1.** PCA plots of PDGF and TGFB stimulated SMC and Fibroblasts datasets.4 microarray datasets were identified from NCBI GEO database in which fibroblasts or smooth muscle cells were stimulated with PDGF or TGFB. PCA and scree plots were generated using the raw expression data to determine the extent of variation between conditions and replicates. The first plot in each pair of plots contains the first two principle components (PC1 and PC2) on the x and y axis respectively along with the percentage of the total variation they account for in each dataset. The second plot in each of the panels represent all principle components and the percentage of total variation with the dataset that each principle component accounts for ranked from greatest to least variation. A) The dataset, GSE52488, is previously published by us and contains primary Human bladder smooth muscle cells (pHBSMC) stimulated with vehicle control (Ctrl) or PDGF for 24hrs.B) GSE63883 comprises Human airway smooth muscle cells stimulated with vehicle control (Ctrl) or TGFB for 8hrs. C) GSE14256 comprises human fibroblasts stimulated with vehicle control (Ctrl) or PDGF for 24hrs. D) GSE61128 comprises human fibroblasts stimulated with vehicle control (Ctrl) or TGFB for 6hrs. **Supplemental Figure 2.** Secondary TF enrichment analysis using UCSC TFBS. Volcano plots of top 3000 differentially expressed genes in which the log2 fold (log2FC) changes and –log10 transformation of the *p*-values were plotted on the x and y axis respectively. Genes that met the log2FC cutoffs of less than -0.25 or greater than 0.25 as well as an adjusted *p*-value cut off of < 0.05 were colored in red. In green are genes that only meet the log2FC cutoffs, in blue are the genes that only meet the *p*-value cutoffs and the genes in grey meet none of the cutoffs. A) Differentially expressed genes from GSE63383, comprising Human Airway smooth muscle cells stimulated with TGFB vs control (Ctrl). B) Differentially expressed genes from GSE14256, comprising Human foreskin fibroblasts stimulated with PDGF vs control (Ctrl). C) Differentially expressed genes from GSE61128, comprising Human arterial fibroblasts stimulated with TGFB vs control (Ctrl). Genes that met both cutoffs, identified in read in the volcano plots, were compared with differentially expressed genes from our previously published dataset of primary human bladder smooth muscle cells stimulated with PDGF or vehichle control. Roughly 800 differentially expressed genes (DEG) were common between our dataset and each of the three other datasets. These lists of genes were subjected to transcription factor (TF) enrichment analysis using the UCSC transcription factor binding sequence database. Nearly 170 TF were identified for each list of shared DEGs. D) The top shared TFs (y axis corresponding to each dot) were rank ordered by the percentage of genes in the list of DEGs that were calculated to be regulated by that TF (indicated by the size of each dot). The –log10 transformed adjusted *p*-values were reported as the color of each dot and the fold enrichment of each TF were plotted on the x axis. **Supplemental Figure 3.** No Enrichment of cytoskeleton and chromosome related terms using traditional GSEA approach for GSE63383. A) Standard Gene Set Enrichment Analysis (GSEA) was performed on genes shared between our previously published dataset and those from GSE63383 comprising Human airway smooth muscle cells stimulated with PDGF and compared to control. GO terms for molecular function (MF), biological process (BP) and cellular compartment were determined using a running sum statistic on the shared DEG list rank ordered by log2 fold change (log2FC). Enriched terms were than rank ordered based on the ratio of genes in the DEG list that match the total number of genes associated with each term. Each term is colored based in its adjusted *p*-value.*P*-values were adjusted using Bonferroni method. The size of the dot, corresponding to each term indicate the number of genes from each DEG list that map to each term. GSEA curves where plotted for three or four B) MF terms, C) BP terms and D)CC terms that were statistically most significant and or have the greatest absolute value of normalized enrichment score (NES). **Supplemental Figure 4.** Enrichment of Cytoskeleton and chromosome related terms using traditional GSEA approach for GSE14256.A) Standard Gene Set Enrichment Analysis (GSEA) was performed on genes shared between our previously published dataset and those from GSE14256 comprising Human foreskin fibroblasts stimulated with PDGF and compared to control. GO terms for molecular function (MF), biological process (BP) and cellular compartment were determined using a running sum statistic on the shared DEG list rank ordered by log2 fold change (log2FC). Enriched terms were than rank ordered based on the ratio of genes in the DEG list that match the total number of genes associated with each term. Each term is colored based in its adjusted *p*-value.*P*-values are adjusted using Bonferroni method. The size of the dot, corresponding to each term indicate the number of genes from each DEG list that map to each term. GSEA curves where plotted for three or four B) MF terms, C) BP terms and D) CC terms that were statistically most significant and or have the greatest absolute value of normalized enrichment score (NES). **Supplemental Figure 5.** No Enrichment of cytoskeleton and chromosome related terms using traditional GSEA approach for GSE61128. A) Standard Gene Set Enrichment Analysis (GSEA) was performed on genes shared between our previously published dataset and those from GSE61128 comprising Human aortic fibroblasts stimulated with TGFB and compared to control. GO terms for molecular function (MF), biological process (BP) and cellular compartment were determined using a running sum statistic on the shared DEG list rank ordered by log2 fold change (log2FC). Enriched terms were than rank ordered based on the ratio of genes in the DEG list that match the total number of genes associated with each term. Each term is colored based in its adjusted *p*-value. *P*-values are adjusted using Bonferroni method. The size of the dot, corresponding to each term indicate the number of genes from each DEG list that map to each term. GSEA curves where plotted for three or four B) MF terms, C) BP terms and D) CC terms that were statistically most significant and or have the greatest absolute value of normalized enrichment score (NES). **Supplemental Figure 6.** Enrichment analysis using GO-CC terms; changes in chromatin remodeling and cytoskeleton. Shared DEGs were identified between our PDGF stimulated pHBSMC dataset and each of the three other datasets. These DEG lists, along with the log2 fold change and *p*-values for each gene were subjected to active subnetwork searches against the BIOGRID protein-protein interaction network followed by gene ontology enrichment analysis for terms related to cellular compartment. Similar terms were then clustered. These analyses were performed using functions from the pathfindR package. In all comparisons of our dataset, either with A)GSE63383 in which smooth muscle cells were stimulated with TGFb, B) GSE14256 in which fibroblasts were stimulated with PDGF, or C) GSE61128 in which fibroblasts were stimulated TGFb, there was an enrichment of terms related to chromatin (clusters highlighted with orange boxes) and for changes in the cytoskeleton (clusters highlighted with green boxes). **Supplemental Figure 7.** Identification of shared genes between all 4 datasets, TF regulator analysis and enrichment analysis. Differentially expressed genes (DEGs) form each of the four microarray datasets were compared via A) a 4 way Venn-diagram to identify the number of genes that were common to all four datasets. B) MYC and MAX were among the top TF predicted to regulate these shared genes based on -log Fisher's exact test (FET) *p*-value. Enrichment analysis of the shared genes was performed using enrichGo function from the clusterprofiler R package without the use of Log2 fold changes or *p*-values searching for C) GO-MF terms, D)GO-BP terms and **E) **GO-CC terms. **Supplemental Figure 8.** Quality assessment of Data sets using PCA plots to determine homogeneity among treatment groups. Quality assessment of publicly available data in which mechanically active cells are stimulated with A)PDGF (GSE111714), B)TGFb (GSE127229) or C) Growth media (GSE138323) with or without JQ1 using PCA plots. More than 80% of the variation for each dataset was between the conditions and not within the replicates as captured in the first two principle components. **Supplemental Figure 9.** Enrichment analysis of JQ1 sensitive genes using GO-MF terms. JQ1 sensitive genes for each dataset were identified with the DEGreport package on normalized count data. The JQ1 sensitive genes, regardless of the direction of change in expression were then subject to active subnetwork search against the BIOGRID database of protein-protein interaction networks followed by enrichment analysis and clustering using functions from the pathfindR package. Clusters of enriched GO terms related to molecular function were rank ordered by adjusted *p*-value of the first term in each cluster and plotted as dot plots. A-C)Clusters of terms for chromatin related changes are highlighted in orange and cytoskeleton related changes are highlighted in green. **Supplemental Figure 10. **Pathway and expression of JQ1 sensitive cytoskeleton associated genes from PDGF and TGFB datasets. A) JQ1 sensitive genes from the PDGF and TGFB datasets were filtered for genes in the KEGG pathway, “Regulation of the actin cytoskeleton”. Green genes were perturbed by TGFB and returned near to baseline with JQ1. Red genes were perturbed by PDGF and returned near baseline with JQ1. Red and Green colored genes were perturbed in both datasets and returned to near baseline expression by JQ1. B)The same 39 unique genes were plotted based on their baseline mean expression and log2 fold change. The color of each gene reflects the-log10 transformation of the adjusted *p*-value. Triangles indicate JQ1 sensitive genes from the TGFB dataset. Circles indicate JQ1 sensitive genes from the PDGF dataset. Genes with a mean expression count of greater than 3000 and an absolute log2FC of greater than 0.15 or 20% were labeled and considered for *in vitro *validation.**Supplemental Figure 11.** RNAseq dataset of fibroblasts stimulated with JQ1 or shBRD4 under proliferative or quiescent conditions. RNA-seq dataset comprising human fibroblasts under conditions of growth or quiescence and treated with JQ1, DMSO, shCtrl, or shBRD4 (GSE189585). A) Principle component analysis identified 75% of the total variation in the dataset in the first two principle components was attributed to the difference among the conditions.B) Skree plot of all principle components plotted on the x axis and the percentage of the total variation they account for within the dataset. **Supplemental Figure 12.** Pattern Analysis identifies groups of genes attenuated by JQ1 and/or BRD4. Three conditions were assesed to identify the JQ1 sensitive and shBRD sensitive genes. These conditions included DMSO or control shRNA treated quiescent cells (Ctrl_Qsnt), DMSO or control shRNA treated proliferating cells and JQ1 or shBRD4 treated proliferative (Expt_Pro) cells indicated in pink and light blue respectively. JQ1 and BRD4 sensitive genes were calculated with the DEGreport package and grouped into three categories; genes sensitive to JQ1 and not shBRD4 (indicated with a red boarder), genes sensitive to shBRD4 and not JQ1 (indicated with a light-blue boarder) and genes sensitive to both that change in the same direction (indicated with a purple boarder). **Supplemental Figure 13.** Enrichment analysis using CC and MF terms of BRD4 and JQ1 attenuated DEGs induced to near baseline. JQ1 and shBRD4 sensitive genes (JQ1_attenuated and BRD4_attenuated respectively) were separated based on the change in expression comparing the JQ1 or shBRD4 treated proliferative cells to the DMSO or control shRNA proliferative cells. The upregulated genes are subjected to active subnetwork searches using the BIOGRID database of protein-protein interactions followed by enrichment analysis of these networks of genes using A)GO terms for cellular compartment and B)Molecular function. Enriched terms are plotted on the y axis and their fold enrichments are plotted on the x axis. Terms are plotted as dots whose sizes correspond to the number of genes from the DEG lists that are associated with each term. The color of each dot corresponds to the –log10 transformed *p*-values. **Supplemental Figure 14.** Representative genes of JQ1 on and off target attenuation of cytoskeleton associated genes. A)Comparative analyses via an upset plot of 5 groups of gene A) 569 unique genes in the GO-CC library of terms for stress fiber, focal adhesion, and actin cytoskeleton, which were identified as enriched from genes attenuated by both the JQ1 and shBRD treatments. B) 1312 shared genes attenuated by both JQ1 and shBRD C) 1920 JQ1 sensitive genes attenuated only by JQ1. D) 1574 genes predicted to be JQ1 sensitive perturbed by PDGF in dataset GSE111714 and E) the 1108 JQ1 sensitive genes predicted from the TGFb dataset, GSE127229. 4 groups of shared genes were identified. Group 1 (indicated in dark blue) comprises 1 gene (ITGB1) which is shared between gene groups A, B, D and E and represents a BRD4 and JQ1 sensitive (or on-target) cytoskeleton associated gene that is perturbed by both PDGF and TGFB. Group 2 (indicated in light blue) comprises 12 genes and represents JQ1 sensitive, cytoskeleton-associated genes not identified as JQ1 off-targets (exclusive to JQ1 sensitive genes in GSE189585) or as JQ1 on-targets (genes sensitive to both JQ1 and shBRD4). Group 3 and Group (indicated in red and yellow respectively) represent 8 and 9 JQ1 off-target cytoskeleton-associated genes stimulated in the PDGF and TGFB datasets respectively. An additional 3 genes encoding kinases were assessed do to their relevance for initiating, propagating and facilitating changes to the cytoskeleton. These include PDGFRA, SRC, LIMK1. These 33 genes were visualized in two KEGG pathways that represent the majority of these genes, B)regulation of the actin cytoskeleton, and C) focal adhesion. Genes are color coded based on the group to which they were identified. **Supplemental Figure 15.** HBSMC and RBMC comparison and JQ1 dose and time effect on cellular viability. A)A heatmap of C_t_ values from qPCR data comparing RBMC and HBMC gene expression included ECM, smooth muscle and fibroblast specific gene expression. Higher expression is color coded in blue and lower expression is color coded in red. B)Dose and time response of RBMC stimulated with JQ1. **Supplemental Figure 16.** Validation of JQ1 sensitive cytoskeletal genes in pHBSMC. A heatmap of Log2FC values from qPCR data assessing a time course of Veh, PDGF or TGFb stimulation with or without JQ1. Data are representative of 3 independent trials. **Supplemental Figure 17.** JQ1 robustly reduces transcription of contractile genes human and rat bladder contractile cells. A)Smooth muscle markers were assessed in both the RBMC and pHBSMC via qPCR and B)immunoblots. MYC was also assessed via immunoblot to confirm JQ1 reduced MYC protein expression. **Supplemental Figure 18**. JQ1 inhibits contraction of RBMCs on collagen gels after 16hrs with low cytotoxicity. A) A dose and time response of JQ1 mediated inhibition of RBMC contraction of collagen gels and B)assessment of cell viability using Alamar blue. Bar charts represent the means three independent replicates. Error bars reflect the standard deviation. *p*-values were calculated with student ttest. * *p*-value < 0.05, ** *p*-value < 0.01, *** *p*-value < 0.001. Data are representative of 3 independent trials. **Supplemental figure 19.** Uncropped immunoblots of RBMC time course with PBS, PDGF, TGFB, JQ1, and/or DMSO. Representative blots of lysates from RBMC stimulated with vehicle control (V), PDGF (P), or TGFB (T) concurrently with DMSO or JQ1 for 2, 4, 8, and 16 hrs. Blots were stained for ITGA5, ITGB1, PDGFRB, PDGFRA, phosphorylated and total CFL1, SRC, LIMK1. **Supplemental figure 20.** Uncropped immunoblots of pHBSMC and RBMC with PBS, PDGF, TGFB, JQ1, and/or DMSO. Representative blots of lysates from RBMC and pHBSMC stimulated with vehicle control (V), PDGF (P), or TGFB (T) concurrently with DMSO or JQ1 for 16 hrs. Membranes were stained with antibodies against, MYC, CNN1, SM22a, BACT, and Vim

## Data Availability

The datasets analyzed during the current study are available in the NCBI GEO database link. The accession IDs and links to those data are as follows: Microarray data: GSE52488, GSE61128, GSE19106, GSE63383, GSE14256, RNAseq data: GSE111714, GSE127229, GSE148395, GSE138323, GSE189585, GSE72437. Additional data analyzed during the current study are available from the corresponding author on reasonable request.
