# Supplementary material for: Investigation of the impact of bromodomain inhibition on cytoskeleton stability and contraction
Source: Cell Commun Signal. 2024 Mar 16;22:184. doi: 10.1186/s12964-024-01553-6 (PMC10944605; doi:10.1186/s12964-024-01553-6)
Supplement: Supplementary file 2 — Additional file 2: Supplementary Figure 1. PCA plots of PDGF and TGFB stimulated SMC and Fibroblasts datasets.4 microarray datasets were identified from NCBI GEO database in which fibroblasts or smooth muscle cells were stimulated with PDGF or TGFB. PCA and scree plots were generated using the raw expression data to determine the extent of variation between conditions and replicates. The first plot in each pair of plots contains the first two principle components (PC1 and PC2) on the x and y axis respectively along with the percentage of the total variation they account for in each dataset. The second plot in each of the panels represent all principle components and the percentage of total variation with the dataset that each principle component accounts for ranked from greatest to least variation. A) The dataset, GSE52488, is previously published by us and contains primary Human bladder smooth muscle cells (pHBSMC) stimulated with vehicle control (Ctrl) or PDGF for 24hrs.B) GSE63883 comprises Human airway smooth muscle cells stimulated with vehicle control (Ctrl) or TGFB for 8hrs. C) GSE14256 comprises human fibroblasts stimulated with vehicle control (Ctrl) or PDGF for 24hrs. D) GSE61128 comprises human fibroblasts stimulated with vehicle control (Ctrl) or TGFB for 6hrs. Supplemental Figure 2. Secondary TF enrichment analysis using UCSC TFBS. Volcano plots of top 3000 differentially expressed genes in which the log2 fold (log2FC) changes and –log10 transformation of the p-values were plotted on the x and y axis respectively. Genes that met the log2FC cutoffs of less than -0.25 or greater than 0.25 as well as an adjusted p-value cut off of < 0.05 were colored in red. In green are genes that only meet the log2FC cutoffs, in blue are the genes that only meet the p-value cutoffs and the genes in grey meet none of the cutoffs. A) Differentially expressed genes from GSE63383, comprising Human Airway smooth muscle cells stimulated with TGFB vs control (Ctrl). B) Differen [file 12964_2024_1553_MOESM2_ESM.pdf]

Supplemental Figures

Investigation of the impact of bromodomain inhibition on cytoskeleton stability and contraction

Alexander Bigger-Allen[1,2,5], Ali Hashemi Gheinani[1,3,4,5], Rosalyn M. Adam[1,3,5]

[1]Urological Diseases Research Center, Boston Children's Hospital, Boston, MA, USA.
[2]Biological & Biomedical Sciences Program, Division of Medical Sciences, Harvard Medical School, Boston, MA
[3]Department of Surgery, Harvard Medical School, Boston, MA, USA.
[4]Functional Urology Research Group, Department for BioMedical Research DBMR, University of Bern, Switzerland
[5]Broad Institute of MIT and Harvard, Cambridge, MA, USA
Running head: Myc inhibition impacts the cytoskeleton and contraction
Keywords: PDGF, TGFβ, Myc, JQ1, smooth muscle cells, fibroblasts

Correspondence:
Rosalyn M. Adam, PhD,
Urological Diseases Research Center,
Enders Bldg 1061.4,
Boston Children's Hospital,
300 Longwood Avenue,
Boston, MA 02115, USA
E-mail: rosalyn.adam@childrens.harvard.edu
Tel: (617) 919-2019

Conflict of interest: The authors declare that no conflict of interest exists.
Funding acknowledgment: National Institutes of Health R01 DK077195, R01 DK104641, R01 DK127673, and the Children's Urological Foundation.

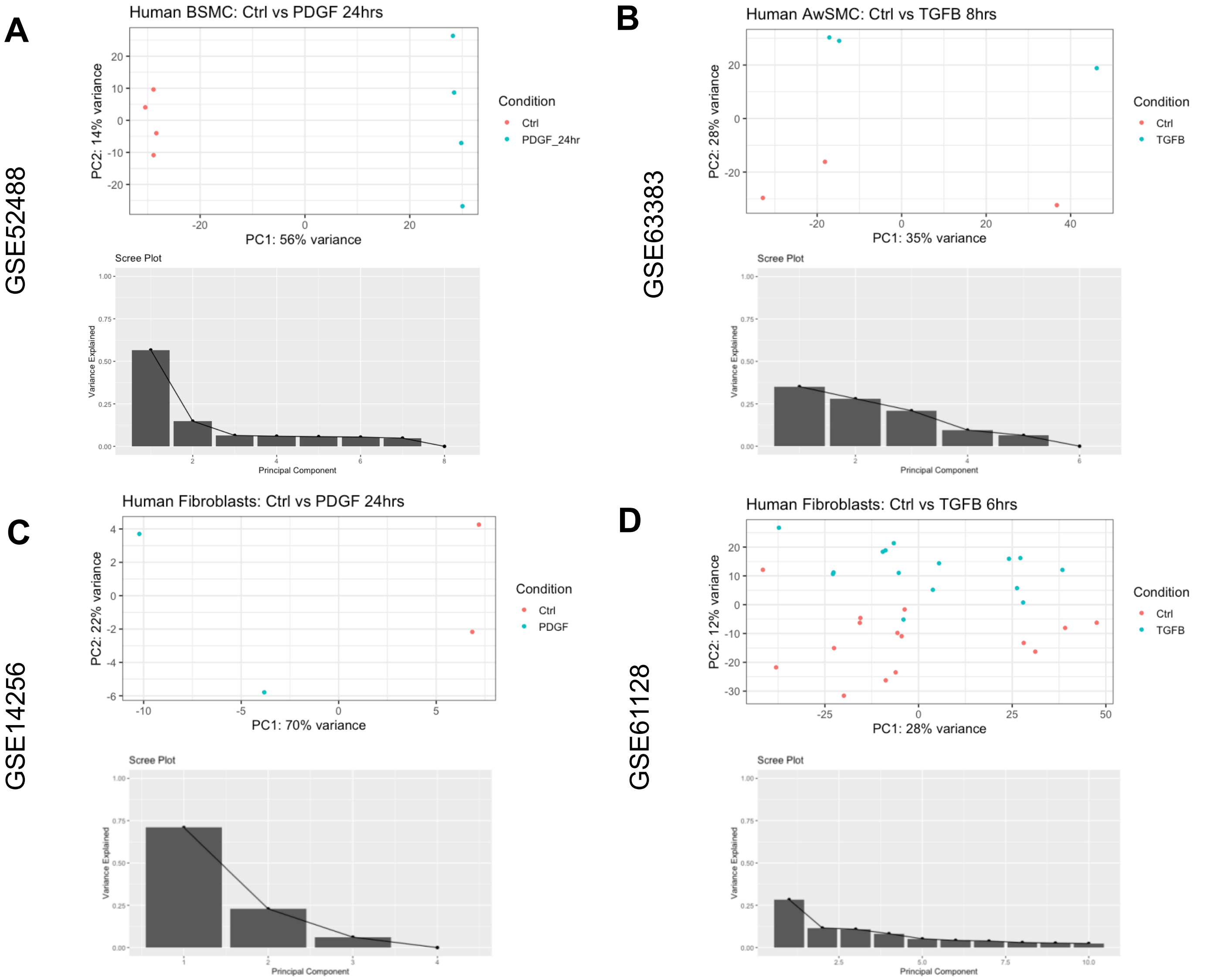

Supplementary Figure 1: PCA plots of PDGF and TGFB stimulated SMC and Fibroblasts datasets

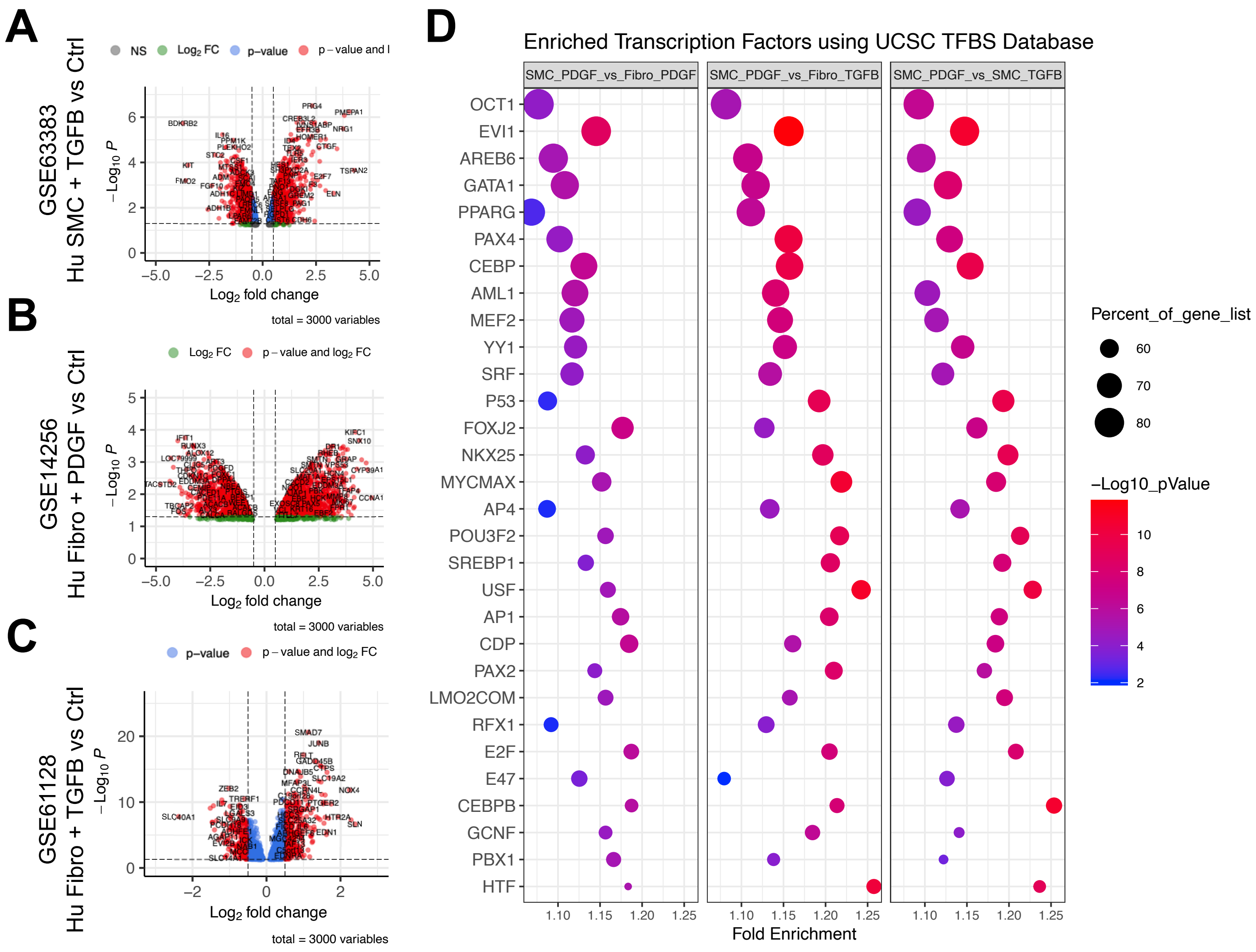

Supplementary Figure 2: Secondary TF enrichment analysis using UCSC TFBS

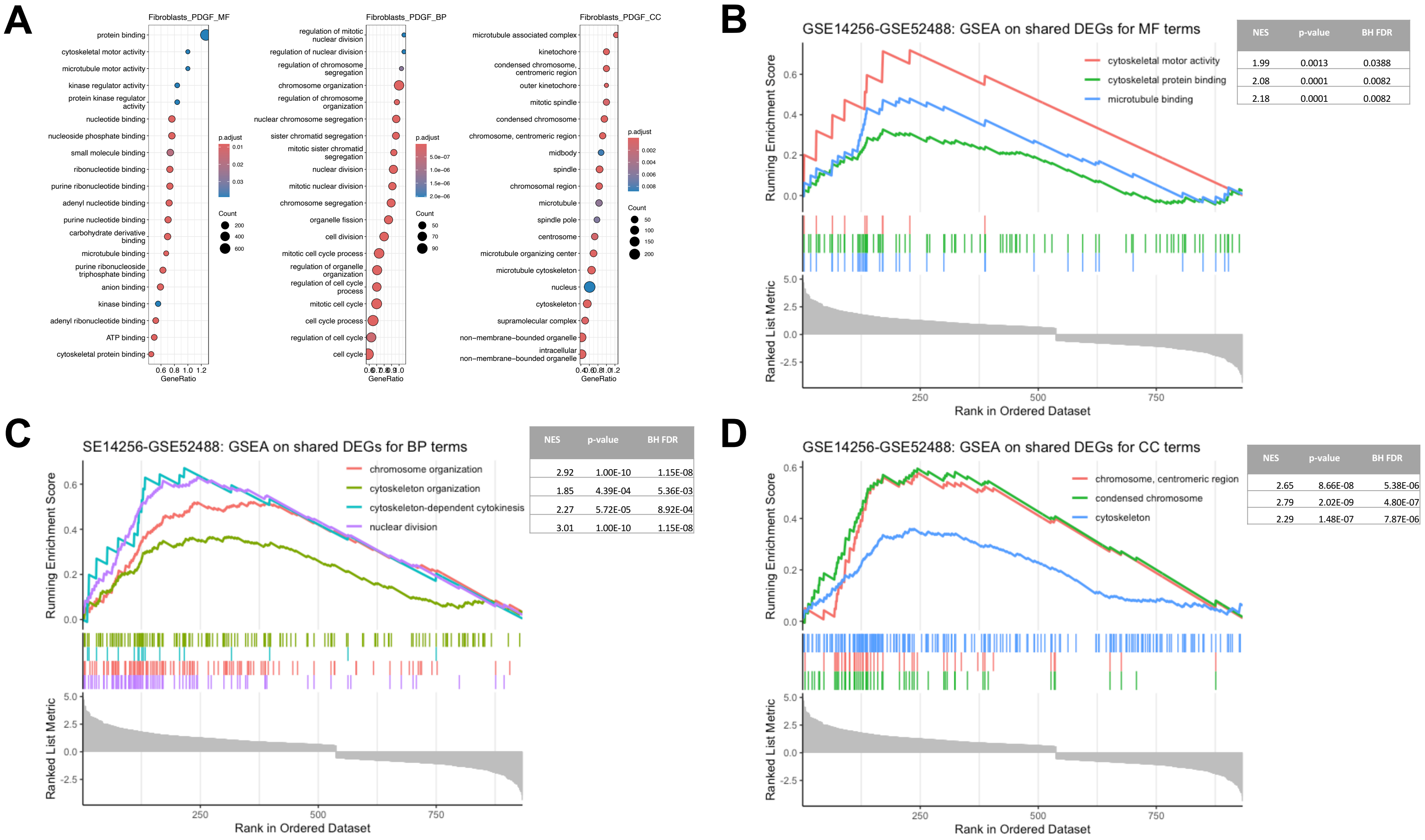

NES	p-value	BH FDR
1.99	0.0013	0.0388
2.08	0.0001	0.0082
2.18	0.0001	0.0082

NES	p-value	BH FDR
2.92	1.00E-10	1.15E-08
1.85	4.39E-04	5.36E-03
2.27	5.72E-05	8.92E-04
3.01	1.00E-10	1.15E-08

NES	p-value	BH FDR
2.65	8.66E-08	5.38E-06
2.79	2.02E-09	4.80E-07
2.29	1.48E-07	7.87E-06

Supplementary Figure 3. Enrichment of Cytoskeleton and chromosome related terms using traditional GSEA approach for GSE14256

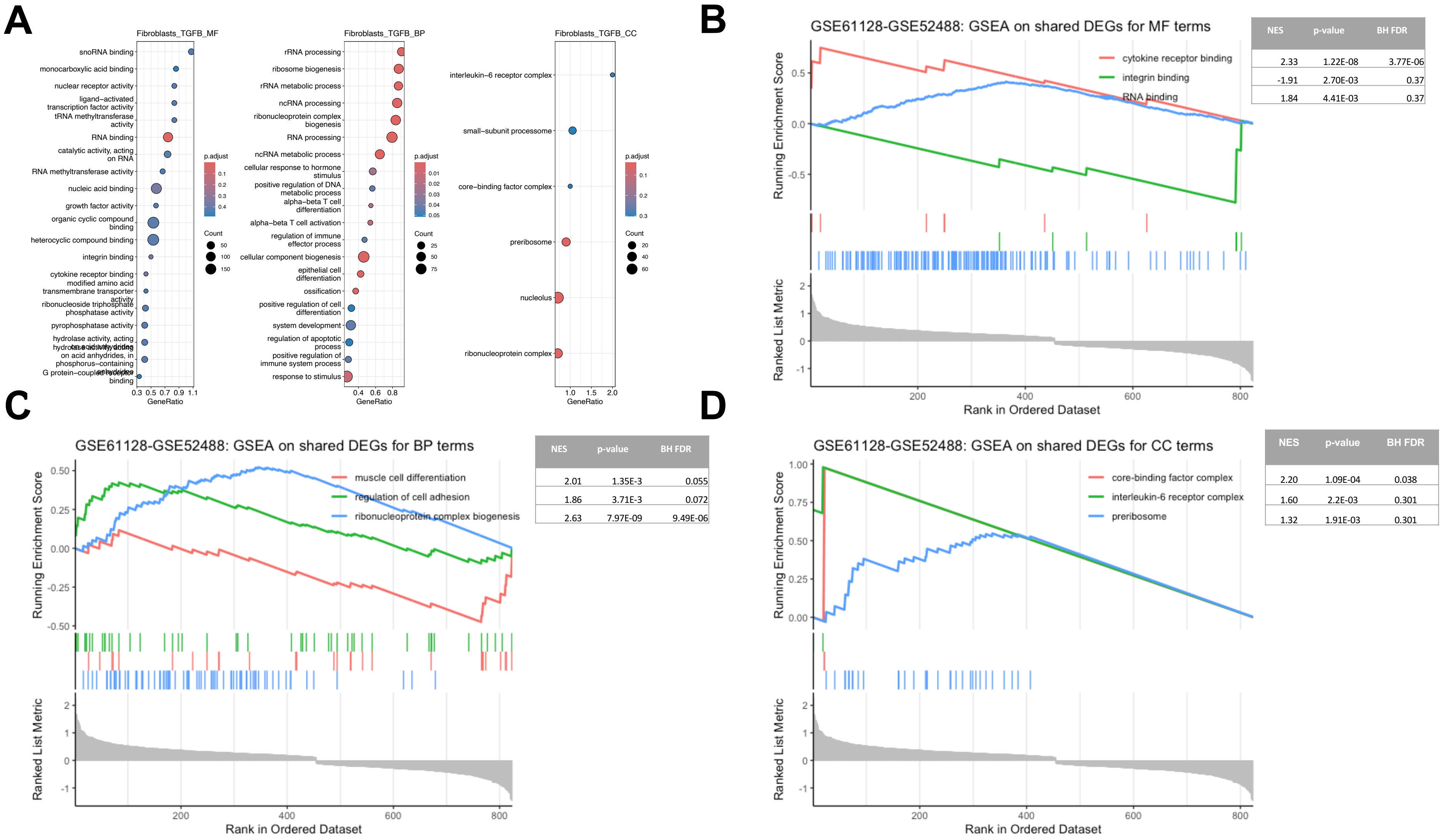

Supplementary Figure 4. No Enrichment of cytoskeleton and chromosome related terms using traditional GSEA approach for GSE61128

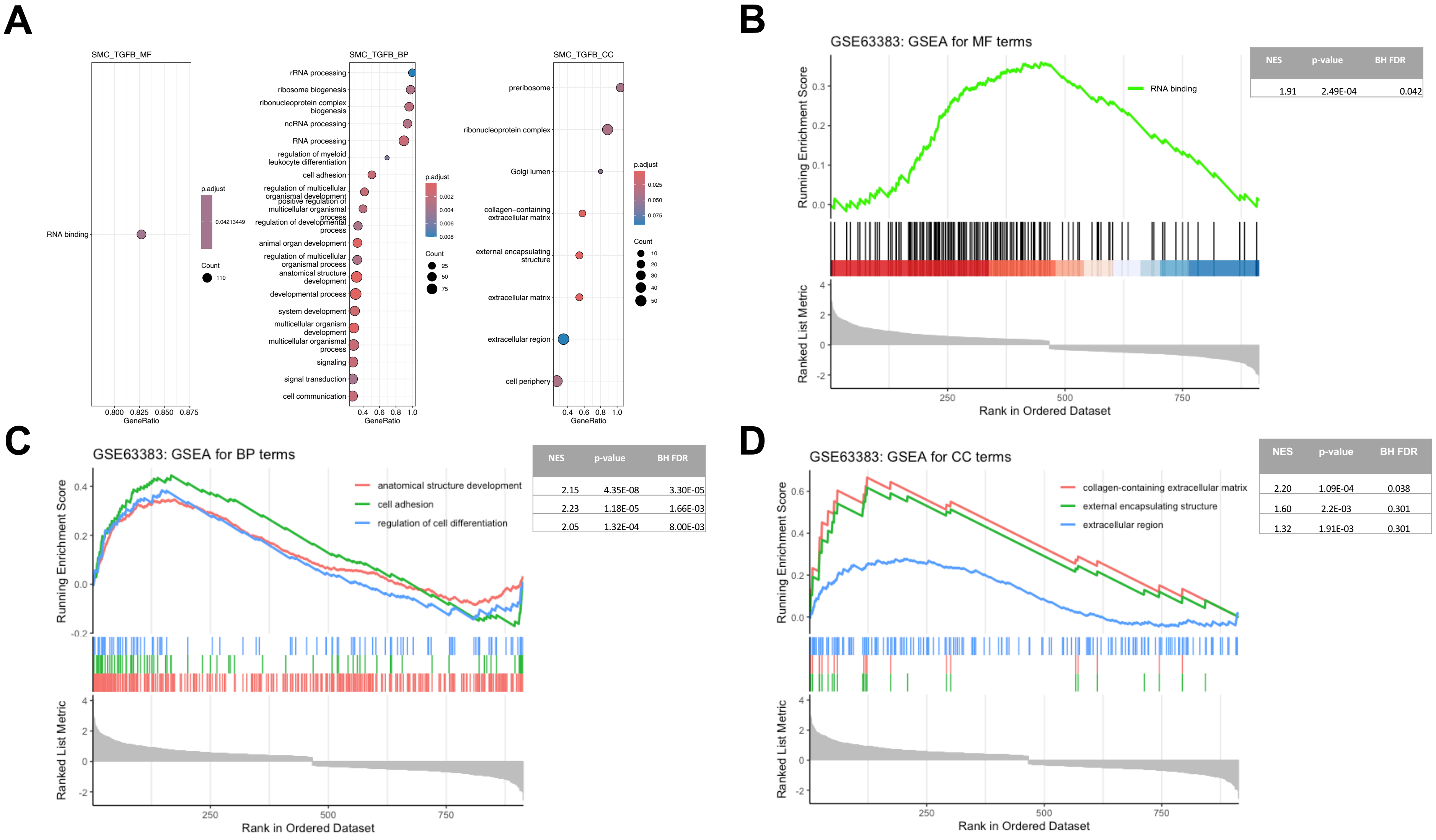

Supplementary Figure 5. No Enrichment of cytoskeleton and chromosome related terms using traditional GSEA approach for GSE63383

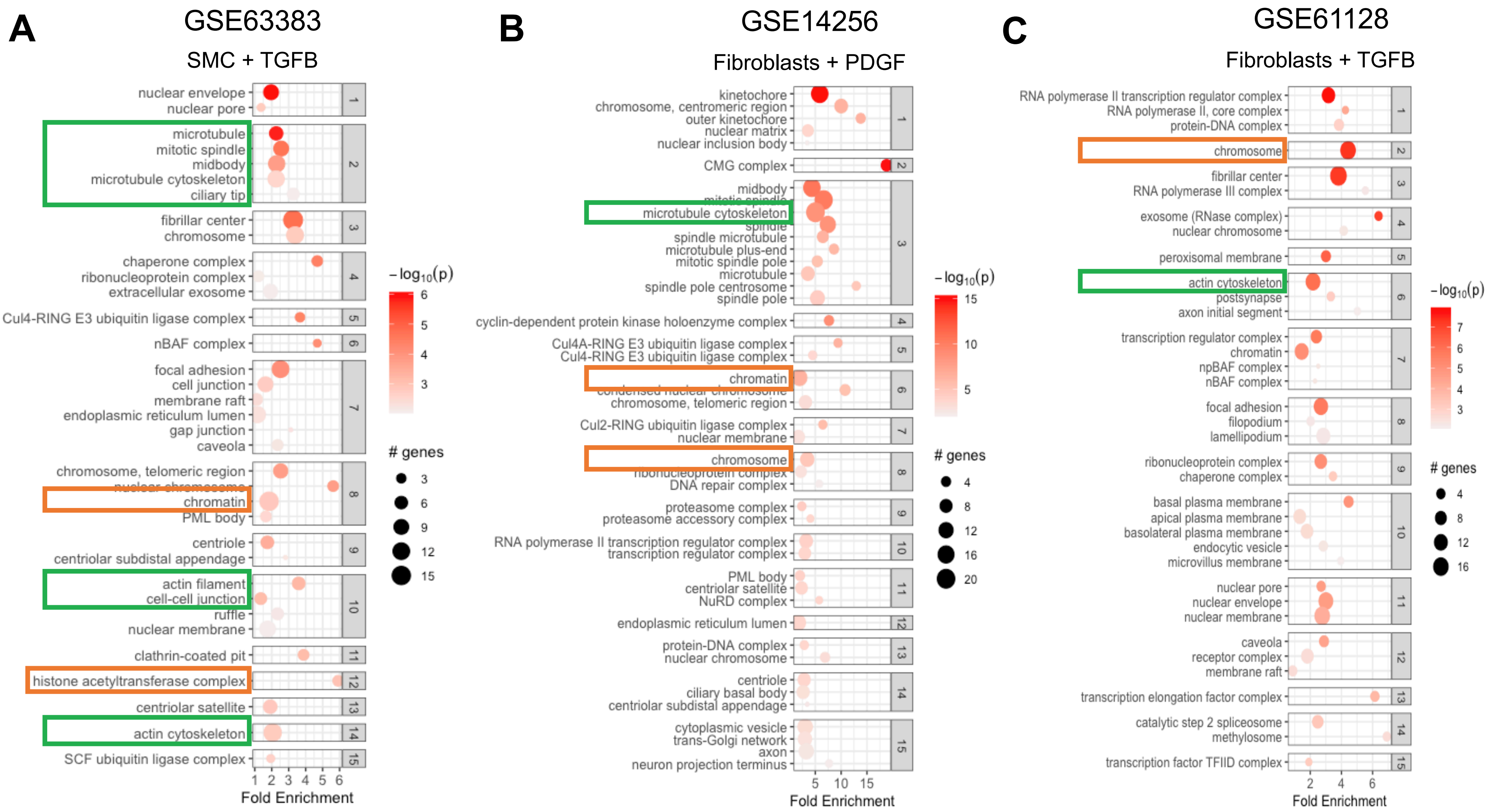

Supplementary Figure 6: Enrichment analysis using GO-CC terms; changes in chromatin remodeling and cytoskeleton

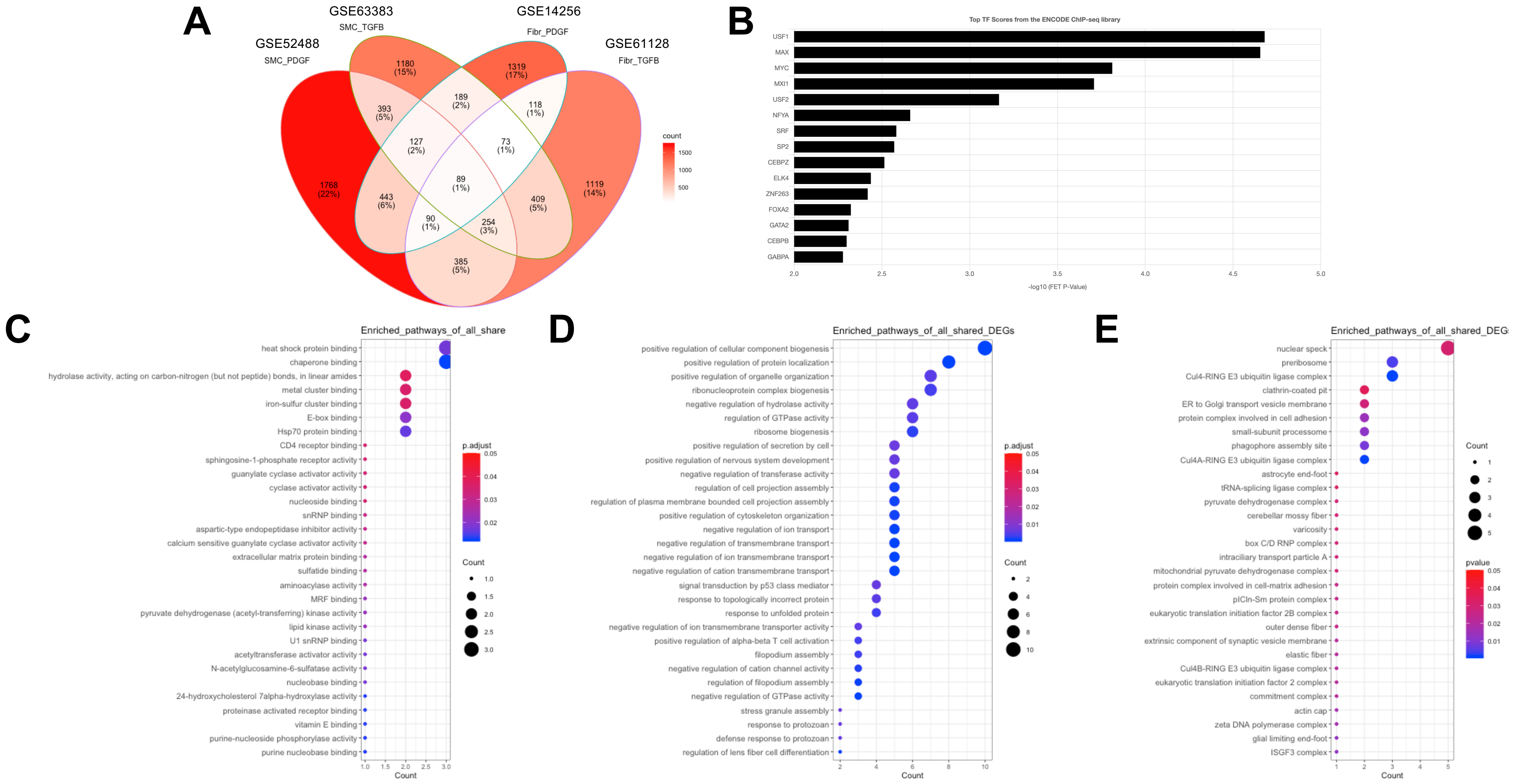

Supplementary Figure 7: Identification of shared genes between all 4 datasets, TF regulator analysis and enrichment analysis

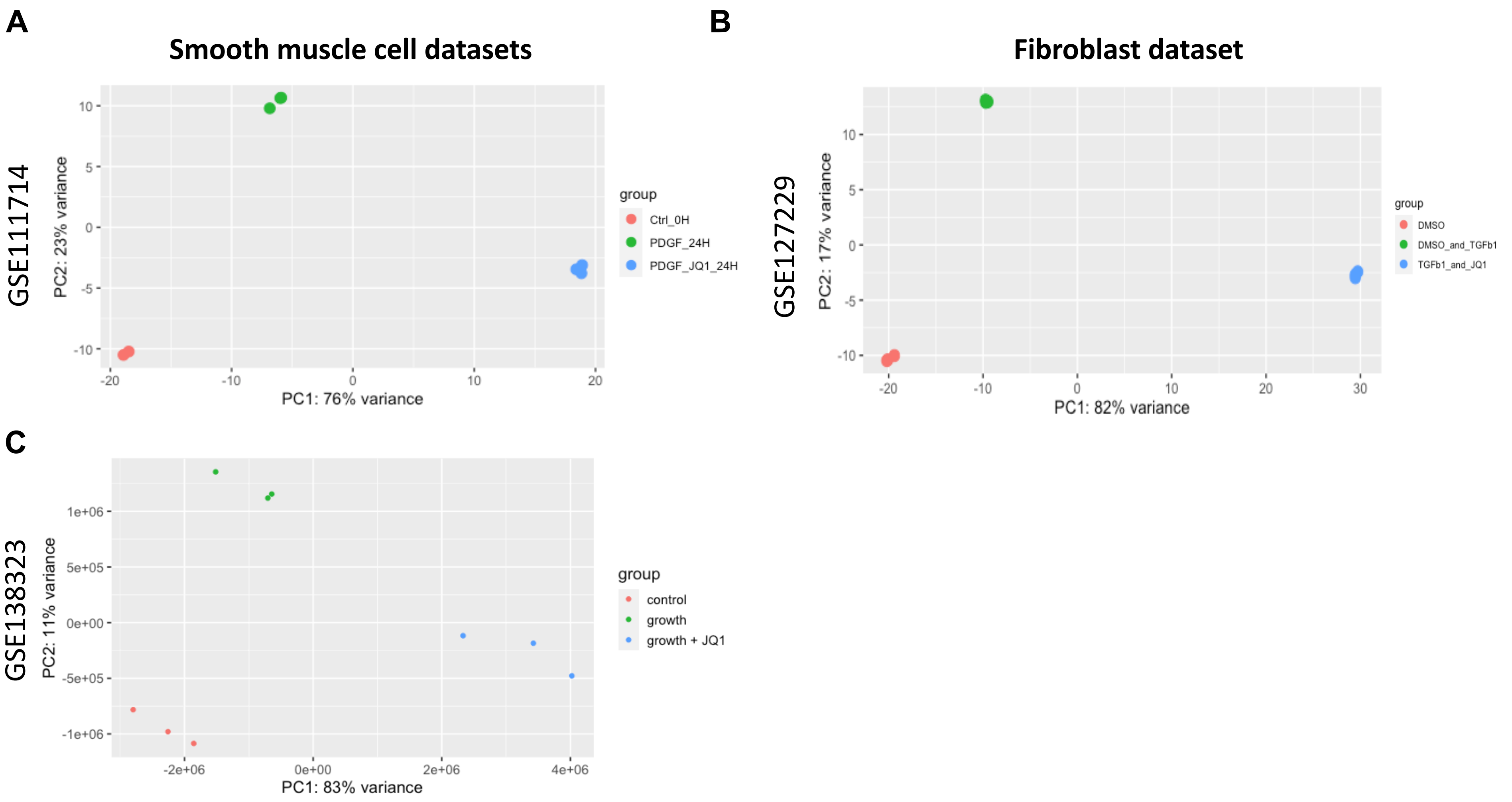

Supplementary Figure 8: Quality control check of Data sets using PCA plots to determine homogeneity among treatment groups

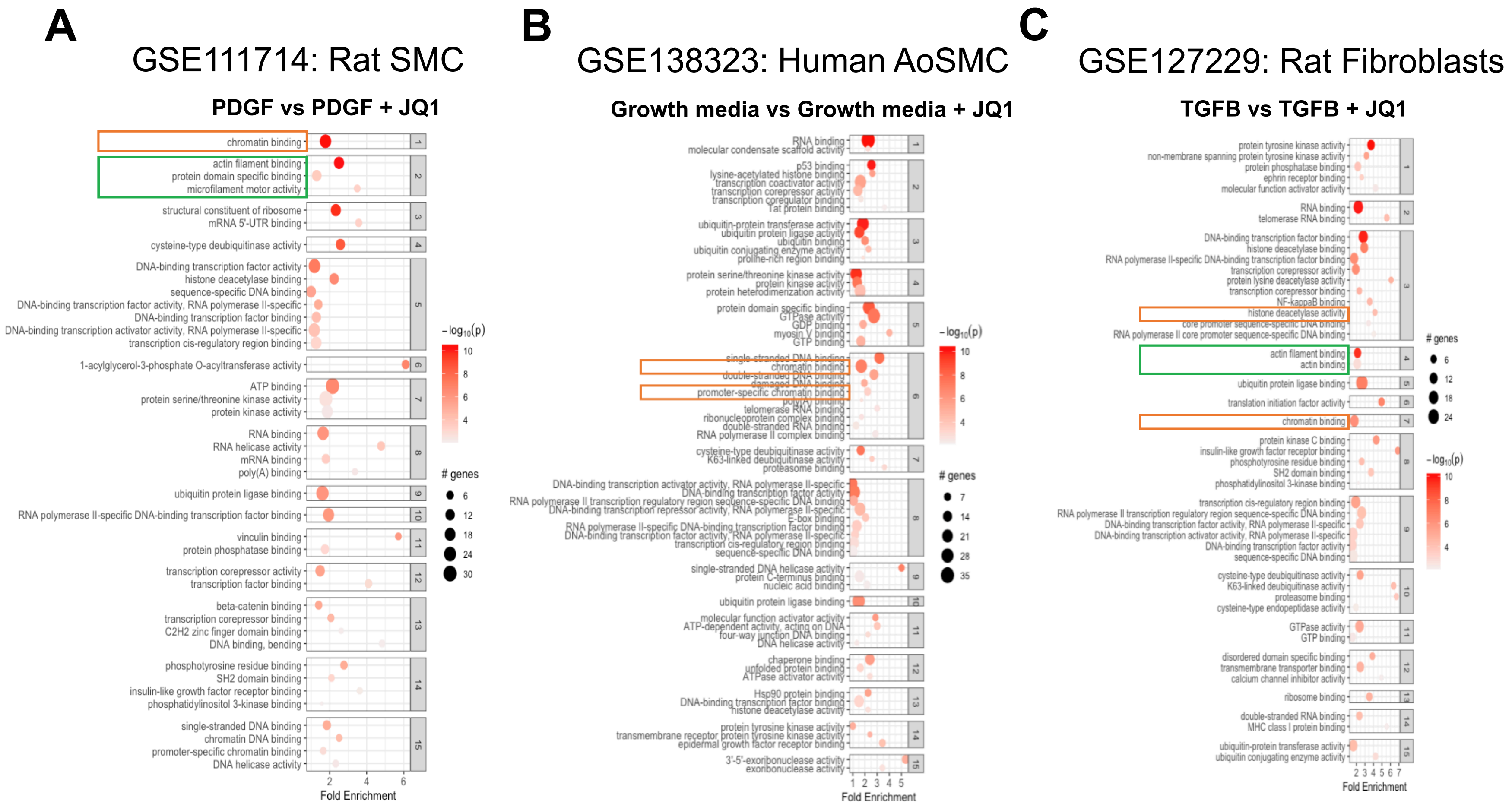

Supplementary Figure 9: Enrichment analysis of JQ1 sensitive genes using GO-MF terms

A

B

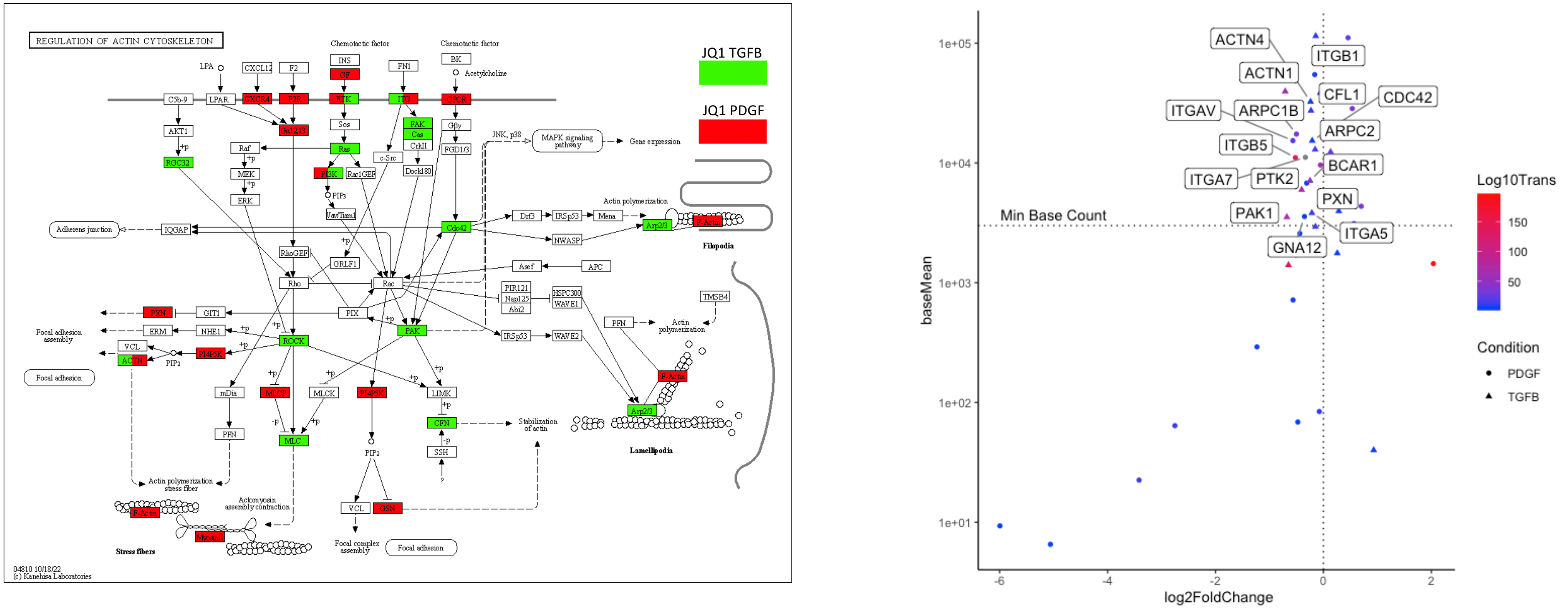

Supplementary Figure 10: Pathway and expression of JQ1 sensitive cytoskeleton associated genes from PDGF and TGFB

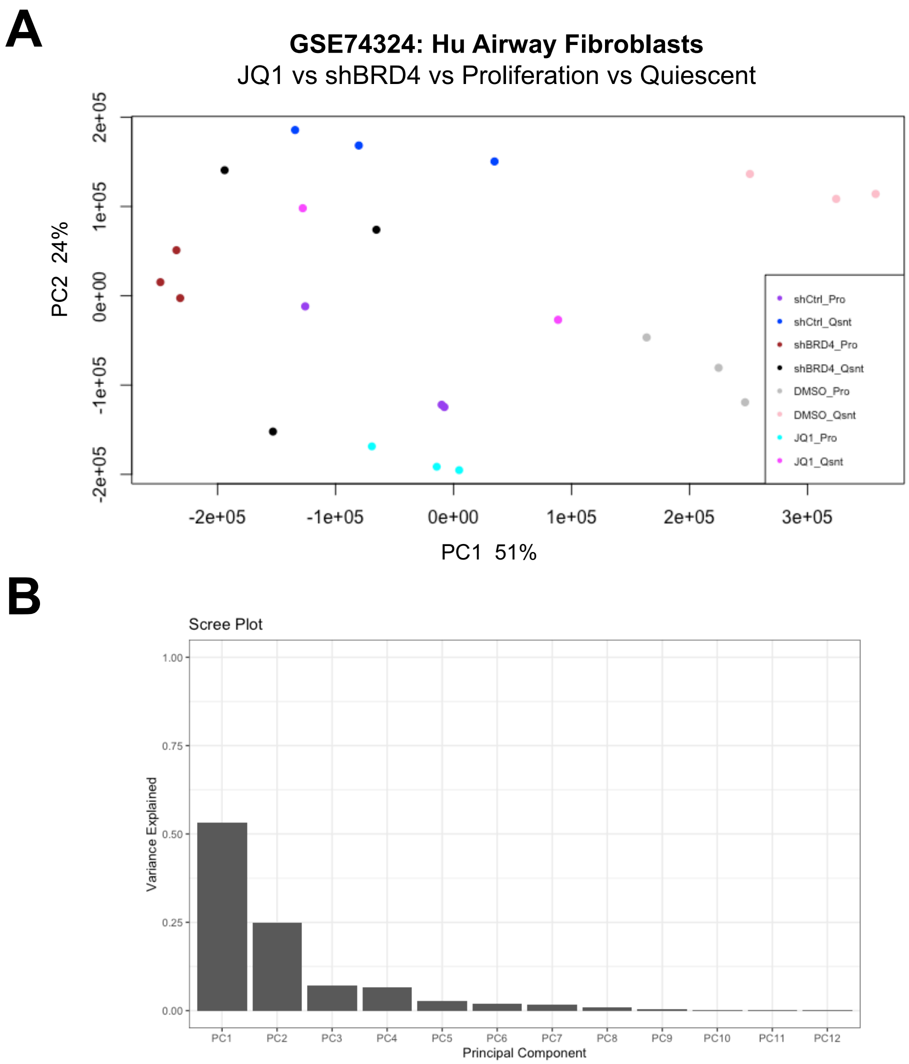

Supplementary Figure 11: RNAseq dataset of fibroblasts stimulated with JQ1 or shBRD4 under proliferative or quiescent conditions

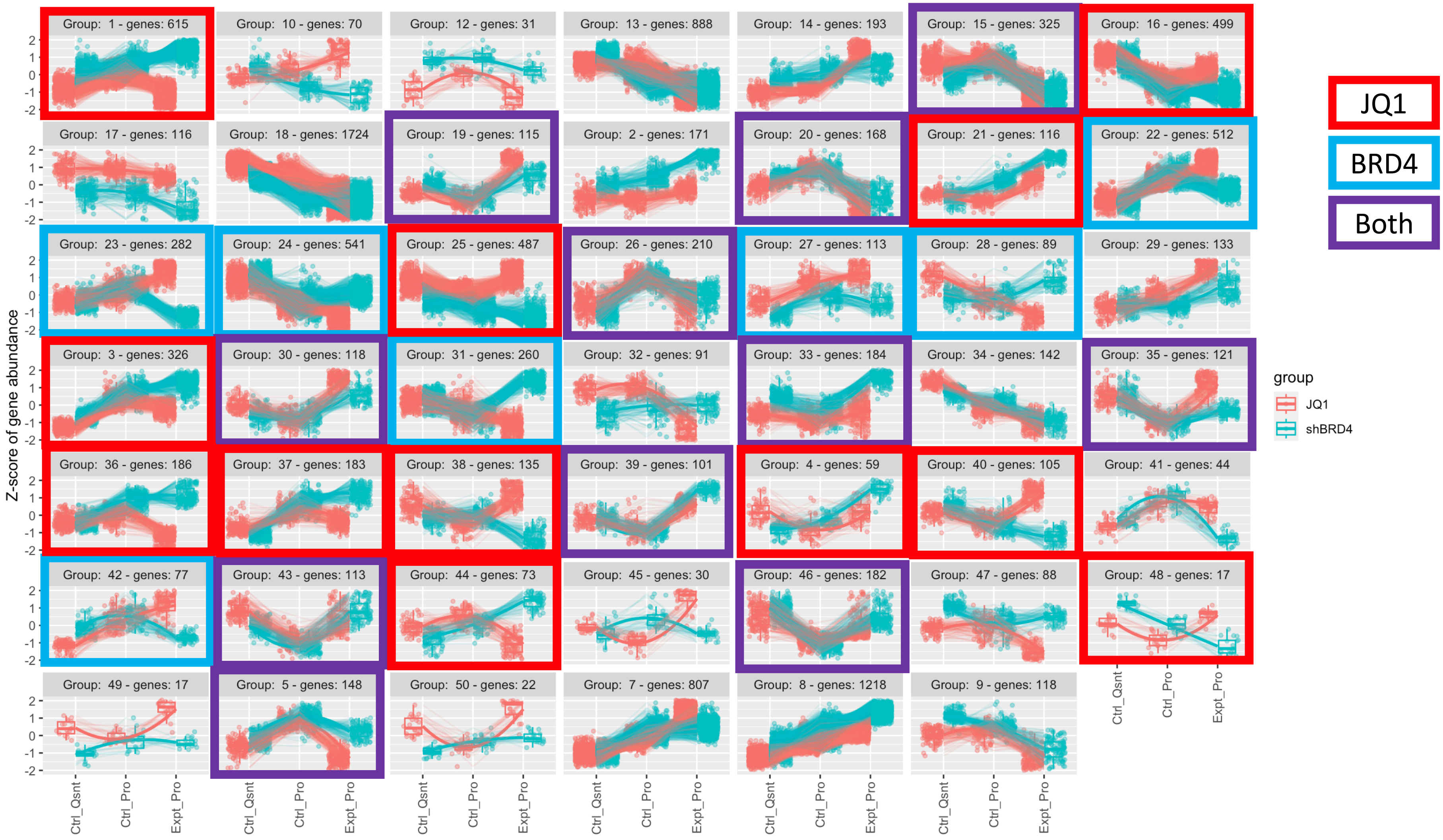

Supplementary Figure 12. Pattern Analysis identifies groups of genes attenuated by JQ1 and/or BRD4

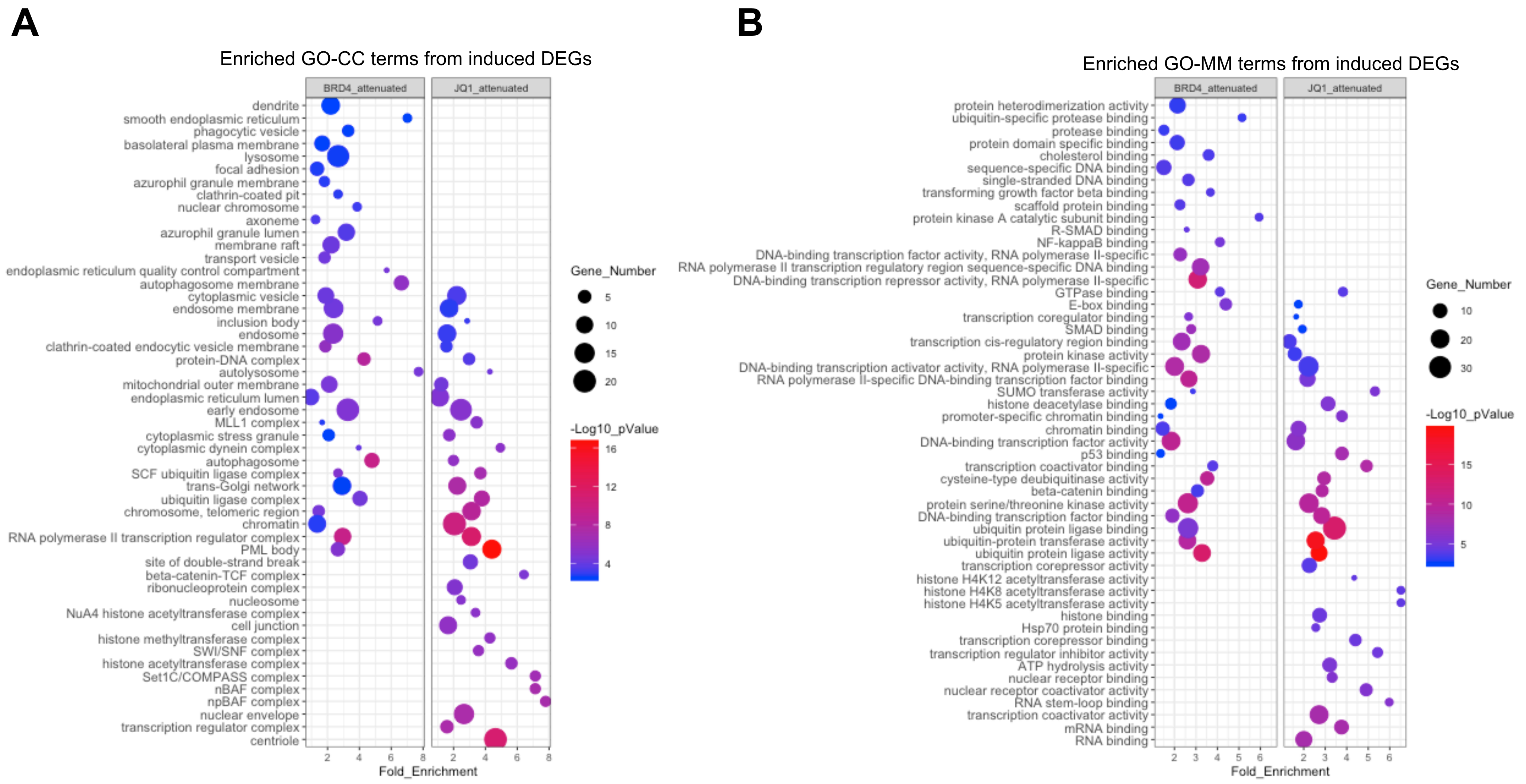

Supplementary Figure 13: Enrichment analysis using CC and MF terms of BRD4 and JQ1 attenuated DEGs induced to near baseline

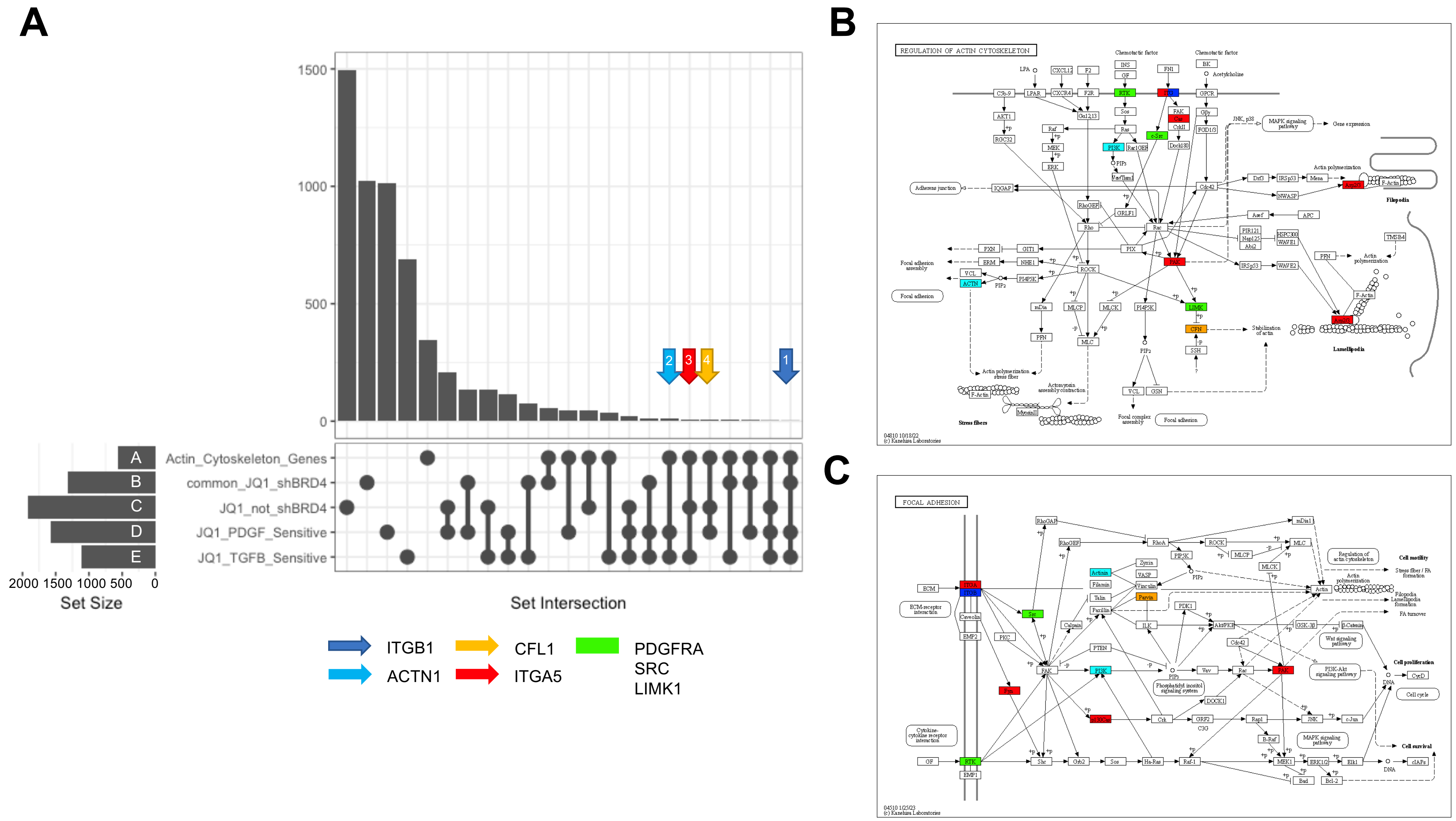

Supplementary Figure 14: Representative genes of JQ1 on and off target attenuation of cytoskeleton associated genes

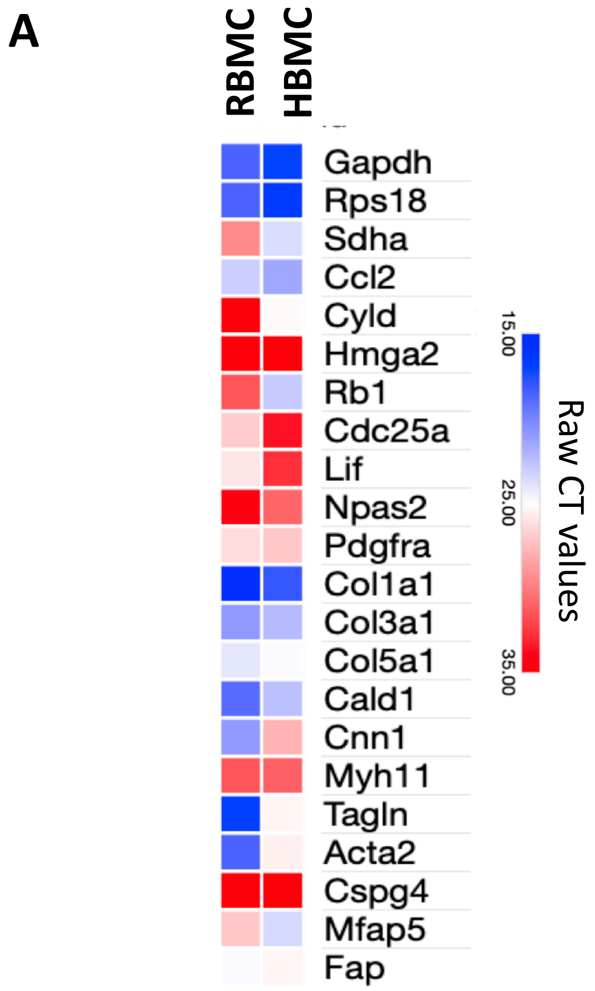

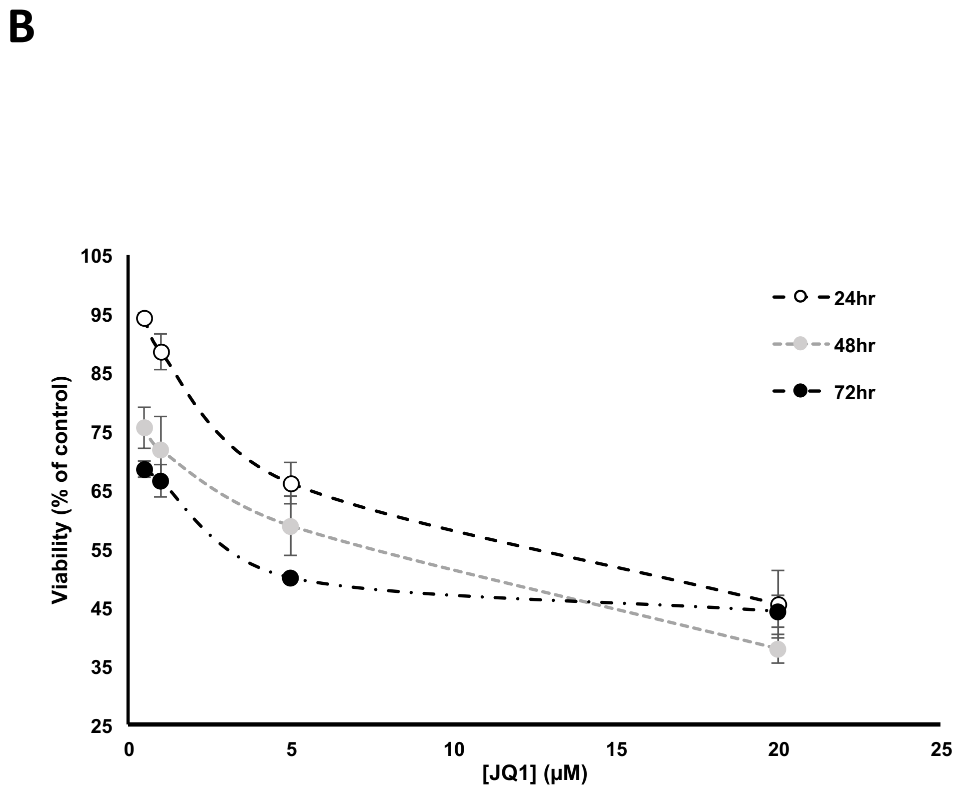

Supplementary Figure 15: HBMC and RBMC comparison and JQ1 dose and time effect on cellular viability

A

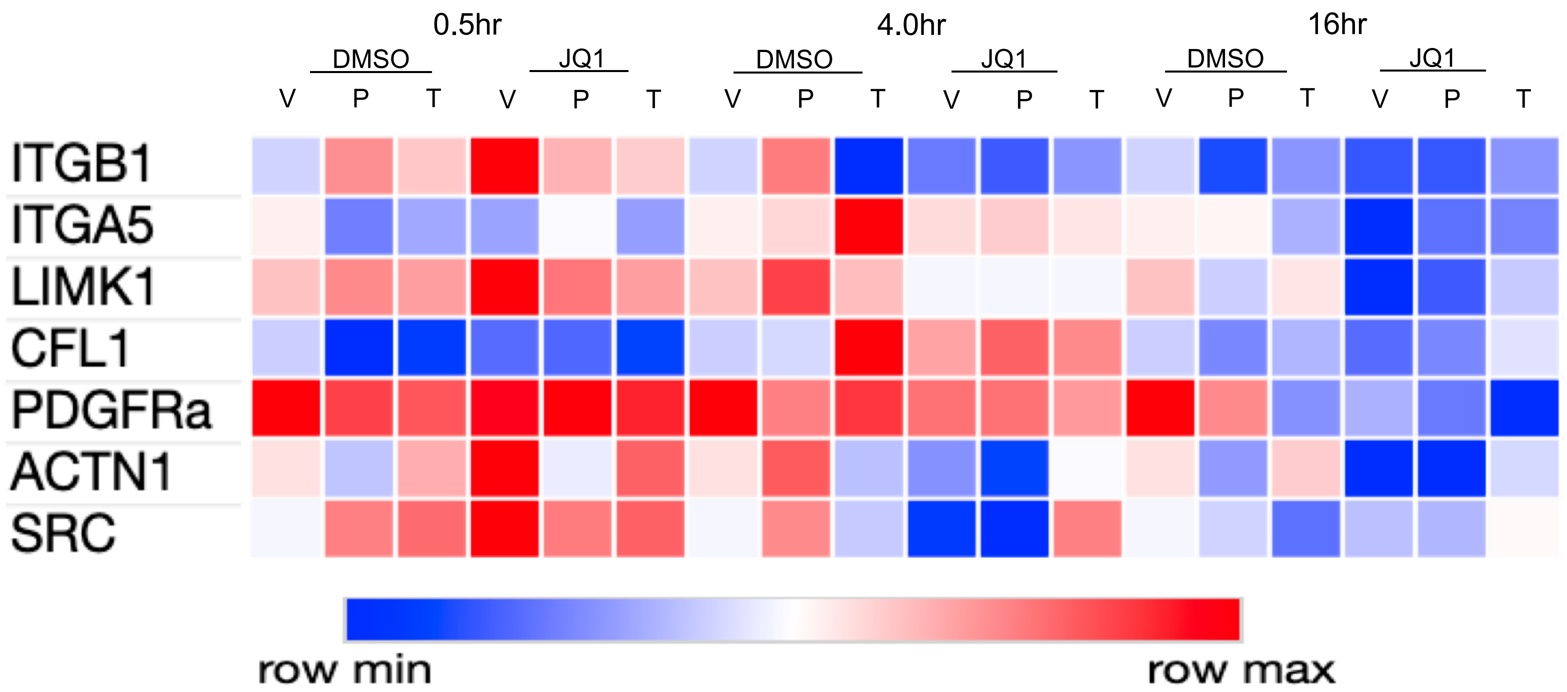

Supplementary Figure 16: qPCR validation of JQ1-sensitive, cytoskeleton-associated gene in pHBSMC

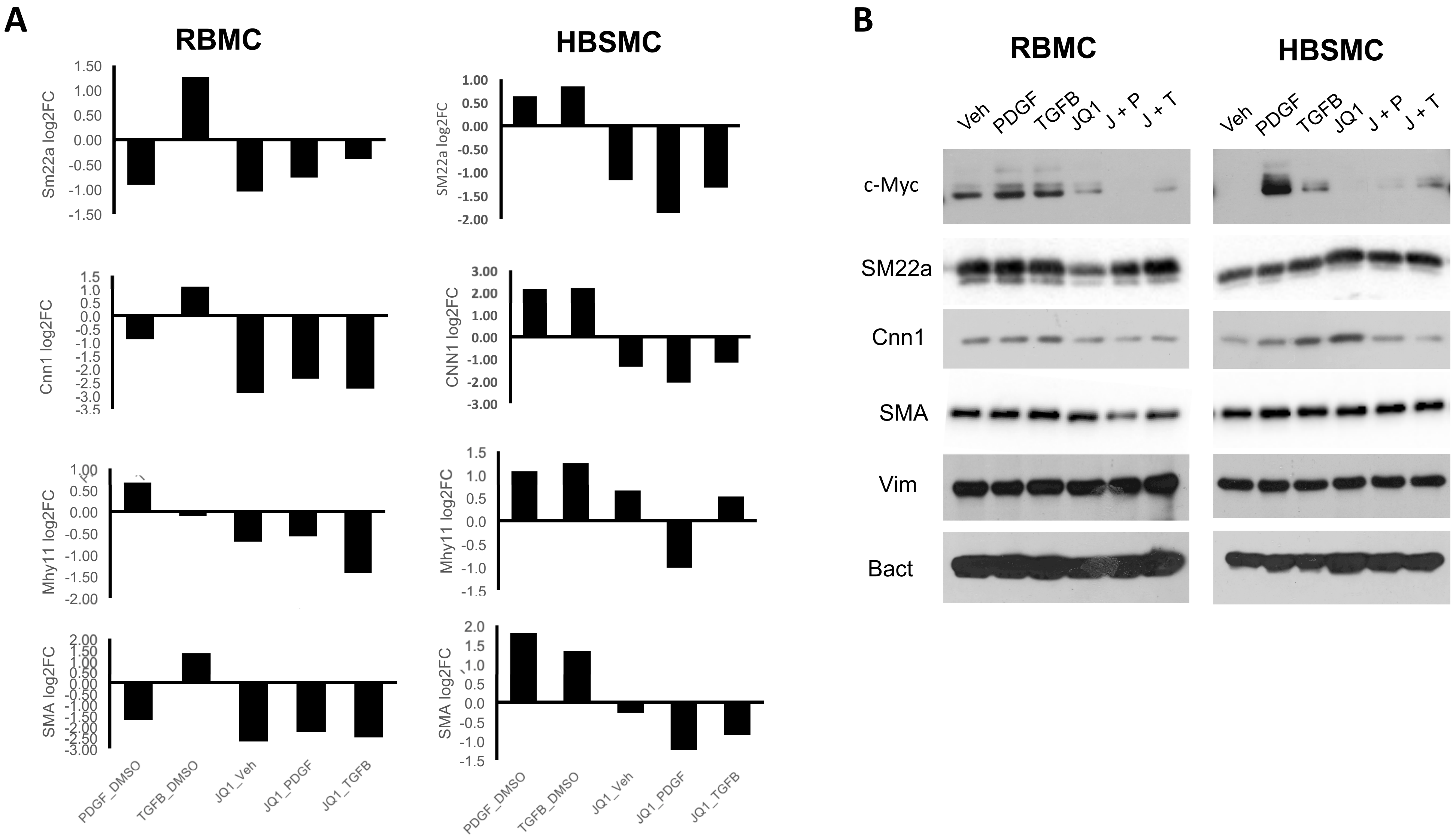

Supplementary figure 17: JQ1 robustly reduces transcription of contractile genes human and rat bladder contractile cells

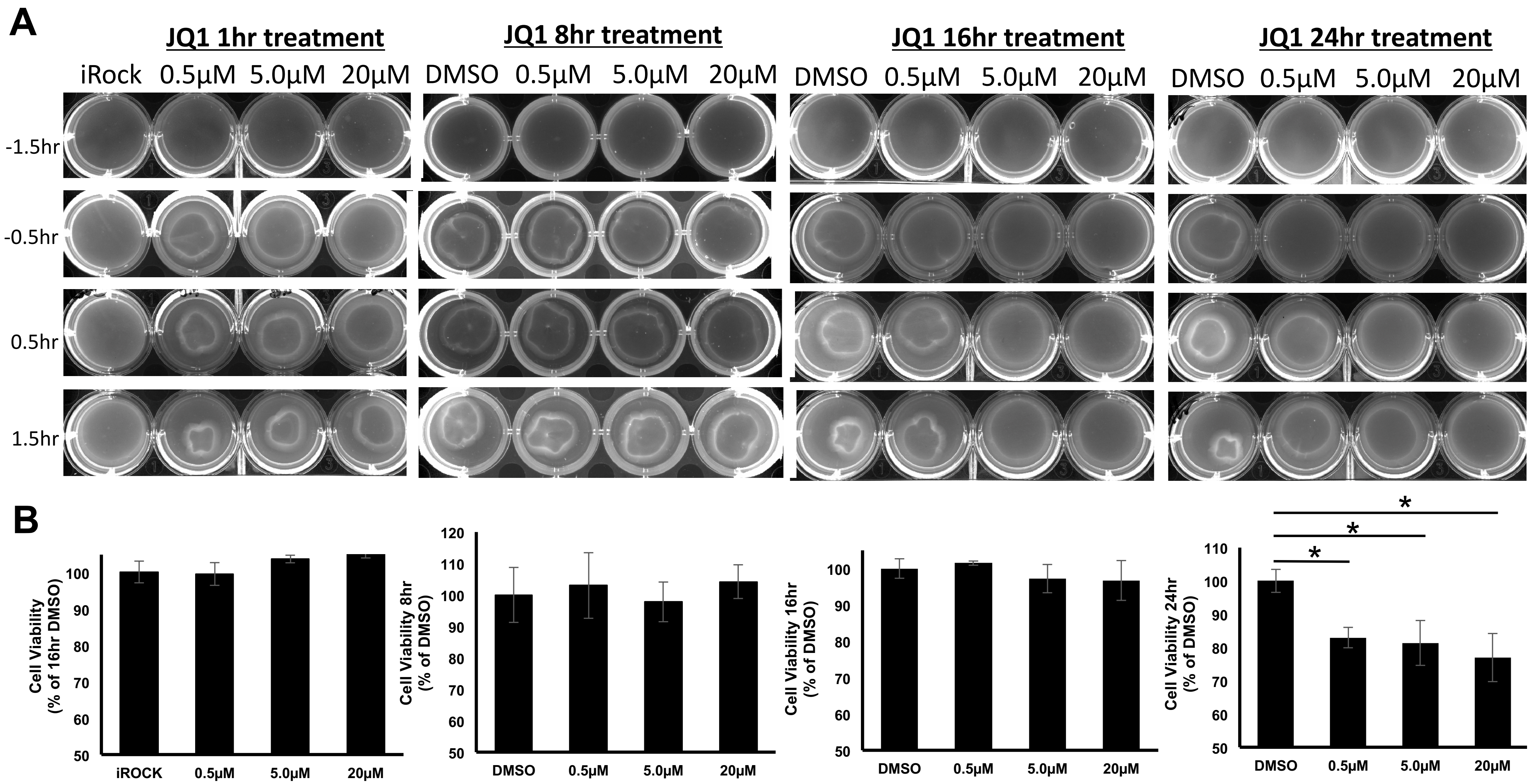

Supplementary figure 18: JQ1 inhibits contraction of RBMCs on collagen gels after 16hrs with low cytotoxicity

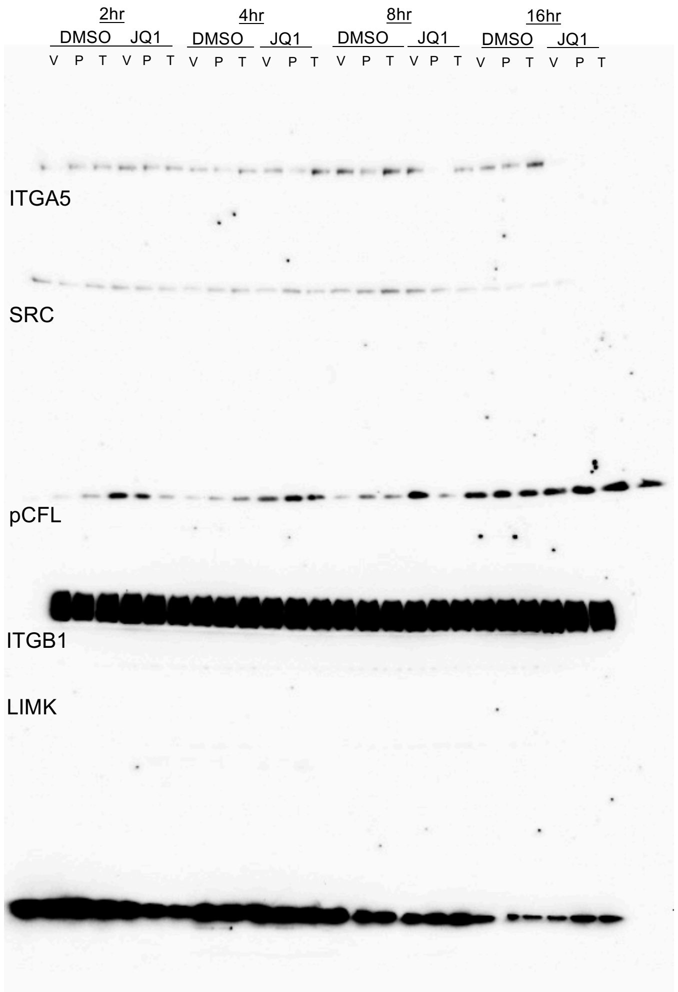

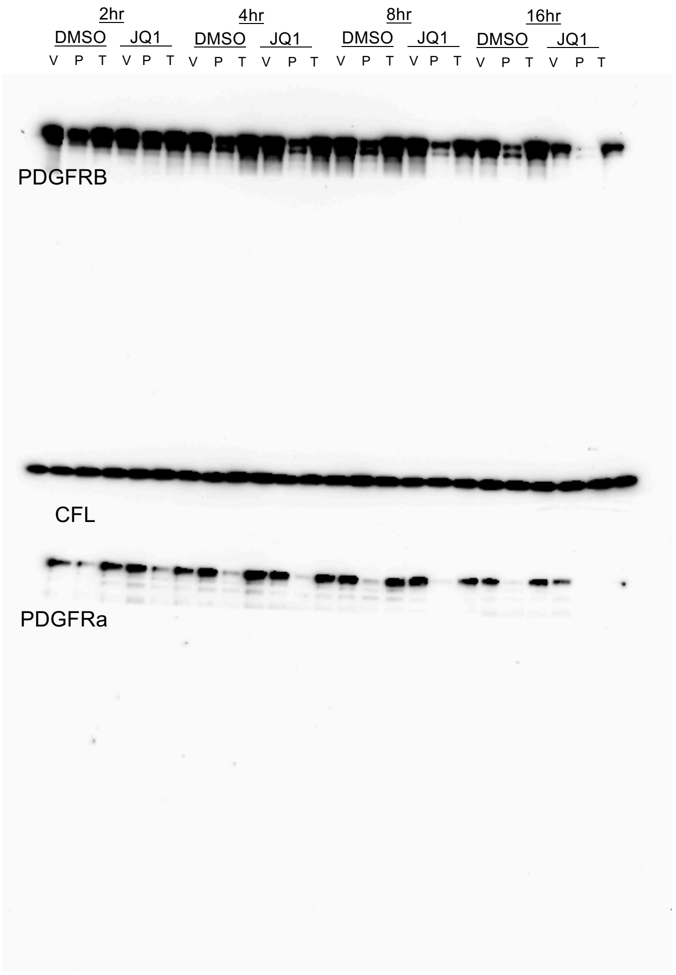

Supplementary figure 19: Uncropped immunoblots of RBMC time course with PBS (v), PDGF, TGFB, JQ1, and/or DMSO

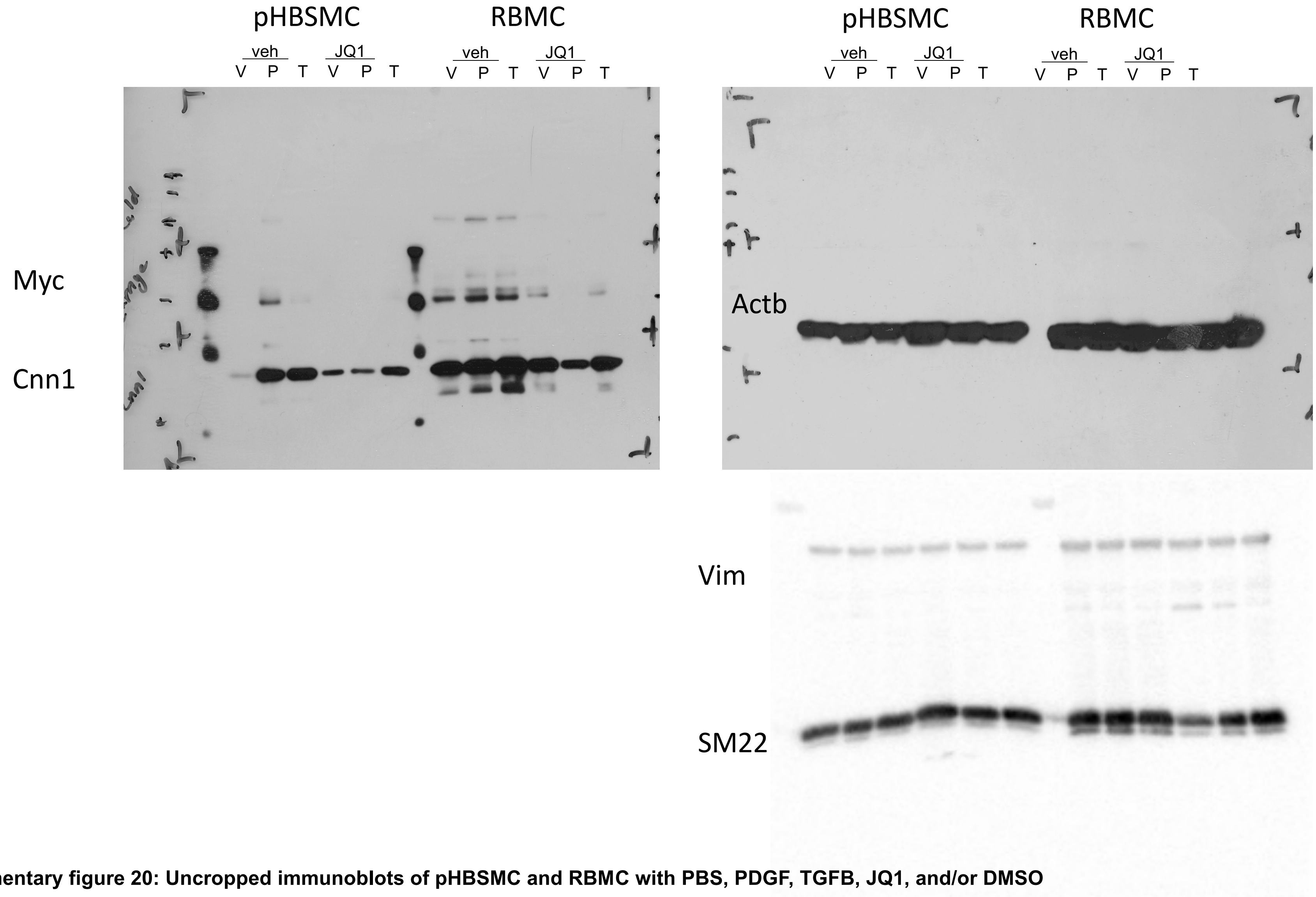

Supplementary figure 20: Uncropped immunoblots of pHBSMC and RBMC with PBS, PDGF, TGFB, JQ1, and/or DMSO
